# The Impact of the United States Foreign Aid Freeze on HIV Service Delivery in PEPFAR‐Supported Countries: A Facility‐Level Analysis of 2024–2025 Programme Data

**DOI:** 10.1002/jia2.70182

**Published:** 2026-07-27

**Authors:** Brian Honermann, Anna Grimsrud, Elise Lankiewicz, Jennifer Sherwood, Greg Millett

**Affiliations:** ^1^ amfAR, Andelson Office of Public Policy Washington USA; ^2^ International AIDS Society Geneva Switzerland

## Abstract

**Introduction:**

On 24 January 2025, the U.S. government issued a stop‐work order freezing foreign assistance, including support for the U.S. President's Emergency Plan for AIDS Relief (PEPFAR), although a limited waiver for selected “life‐saving” interventions was subsequently granted. PEPFAR's official 2025 monitoring results, released 17 April 2026, included only fourth‐quarter data, whereas an earlier inadvertent release contained data for all four quarters of 2025. Using both datasets, we conducted a systematic facility‐level assessment of HIV programme performance while accounting for reporting disruptions.

**Methods:**

Facilities were categorized by reporting between Q1 2024 and Q4 2025 (continuous, intermittent, dropped, new). We assessed changes in HIV treatment, testing, prevention of mother‐to‐child transmission (PMTCT) and HIV prevention across facility categories. We additionally analyse changes in human resources for health (HRH) reported by PEPFAR.

**Results:**

The dataset included 31,628 facilities and community service sites. Most sites reported continuously (71.5%), while 16.7% reported intermittently, 3.9% stopped reporting, 3.1% were new in 2025 and 2.5% were community service sites. Between Q4 2024 and Q4 2025, the total number of people receiving HIV treatment declined by −1%. People on treatment increased 0.3% at continuously reporting facilities but declined −6.0% at intermittently reporting facilities. HIV testing declined by −18% overall. HIV diagnoses decreased by −13% at continuously reporting facilities, −32% in intermittently reporting facilities and −36% in community services sites. PMTCT infant testing declined by −6% at continuously reporting facilities and −60% at intermittently reporting facilities, while infant HIV diagnoses declined by −12% and −31%, respectively. Pre‐exposure prophylaxis initiations declined by −33%, and the number of direct service delivery healthcare workers declined by −62,541 (−24%).

**Conclusions:**

Although HIV treatment remained relatively stable at continuously reporting facilities, substantial declines were observed in HIV testing, HIV diagnoses, treatment initiation, prevention services and the PEPFAR‐supported workforce. These findings suggest that treatment coverage alone is an insufficient indicator of programme resilience. As countries assume greater responsibility for HIV programmes, sustained investment across the entire HIV care cascade coupled with transparent reporting and robust monitoring systems covering the full prevention, testing, treatment and health workforce landscape will be essential to maintain progress towards epidemic control.

## 1 Introduction

The United States President's Emergency Plan for AIDS Relief (PEPFAR), a bilateral programme supporting HIV services in 55 countries, has been credited with saving more than 26 million lives since its inception in 2003 [1]. While PEPFAR's earliest successes centred on prevention of vertical transmission (PMTCT) and initiating antiretroviral therapy (ART) for people living with HIV, by 2024 the programme had expanded to more comprehensive health responses for HIV. It conducted more than 200,000 HIV tests each day, provided services to approximately 17,695 orphans and vulnerable children each day, and supported a total of more than 700,000 people receiving pre‐exposure prophylaxis (PrEP) [2, 3]. PEPFAR investments have been associated with both accelerated epidemic control and measurable health system strengthening in supported countries. Between 2010 and 2023, new HIV infections declined 52% in PEPFAR‐supported settings versus 39% globally, while domestic government health spending and health workforce capacity expanded at nearly double the rate seen in non‐PEPFAR‐supported countries over the same period [1, 4].

In early 2025, however, a series of changes to U.S. foreign policy substantially altered the implementation of PEPFAR. On 20 January 2025, an executive order froze foreign aid disbursements, followed on 24 January by a stop‐work order affecting all foreign assistance awards, including PEPFAR, with limited exceptions for humanitarian assistance [5, 6]. Subsequent waivers allowed the partial resumption of PEPFAR activities, including a programme‐specific waiver permitting a defined subset of HIV care, treatment and prevention of mother‐to‐child transmission services to continue [7]. The following months were marked by considerable instability, including cycles of award cancellations and reinstatements, legal challenges that limited implementation of the freeze by the Centers for Disease Control and Prevention (CDC), and the permanent dissolution of USAID, one of PEPFAR's two major implementing agencies [8]. In response, many country governments reassigned staff and implemented policy and programme changes to maintain continuity of HIV services where possible [9].

The full implications of the foreign aid review were initially difficult to assess because the U.S. government did not release an official list of terminated and active awards following the review. Early assessments, therefore, relied on leaked lists that were incomplete or outdated. Analyses of these lists suggested that 71% of HIV‐focused USAID awards had been cancelled by number, although these represented an estimated 24% of planned PEPFAR funding by value [10, 11, 12]. The potential consequences of these funding disruptions were initially explored through modelling studies rather than observed programmatic data. Under a 90‐day freeze, Hontelez et al. estimated 60,000–74,000 excess HIV deaths across seven sub‐Saharan African countries [13]. Other modelling studies projected that complete or near‐complete discontinuation of PEPFAR could result in an additional 4.4–10.75 million HIV infections and up to 4.1 million HIV‐related deaths by 2030 compared with the status quo [14, 15].

Empirical studies subsequently complemented these modelling studies, demonstrating an initial period of severe disruption, followed by uneven recovery. Immediately after the funding freeze, 71% of PEPFAR implementing partners reported cancelling at least one service category, and 86% anticipated that clients would lose access to ART within a month [16]. As waivers were implemented, some clinical services, particularly HIV treatment, resumed in many countries. However, community‐led services, PrEP, HIV testing and programmes for key populations continued to experience substantial disruption. Similar patterns were observed in other assessments. The IeDEA consortium reported that 47% of clinics across 32 countries continued to experience service disruptions by mid‐2025, while the UNAIDS country‐level impact tracker identified the greatest effects on community‐based services despite continued access to ART in many settings [17, 18].

Until 17 April 2026, however, PEPFAR Monitoring, Evaluation, and Reporting (MER) data for 2025, the most direct measure of PEPFAR programmatic performance, had not been officially publicly released. The only available cross‐country analysis using observed PEPFAR programme data relied on draft MER data obtained outside of official channels and examined only aggregated global trends. That analysis found substantial declines in HIV testing, viral load monitoring and net ART coverage immediately following the policy changes in early 2025, with partial but incomplete recovery by the end of 2025 [19]. However, it did not comprehensively assess the available data or examine facility‐level patterns.

Using the subsequently released full 2025 MER dataset, we conducted a systematic, facility‐level assessment of PEPFAR programme performance and reporting continuity during this period. Specifically, we examined reporting patterns across facilities and community service sites and quantified changes in HIV treatment, testing, PMTCT, prevention and human resources to provide a more comprehensive assessment of the programme impacts associated with the 2025 policy changes.

## 2 Methods

### 2.1 Data Sources and Study Period

We used two versions of PEPFAR MER data: an unofficial dataset released on the PEPFAR development dashboard in January 2026 [19] (“unofficial dataset”) and the official 2025 MER dataset released on 17 April 2026 (“official dataset”) [20].

PEPFAR follows the U.S. government's fiscal year (FY), with Q1 (October−December), Q2 (January−March), Q3 (April−June) and Q4 (July−September). The major policy disruptions in early 2025 occurred during FY 2025 Q2. Officially released 2025 data only included Q4 data, whereas the earlier (unofficial) dataset included all four quarters.

Original datasets for both the unofficial and official dataset releases, as well as processed data, Supplementary Materials and analyses, are available on GitHub [21].

### 2.2 Analytic Approach and Unit of Analysis

PEPFAR's data do not report true zero values distinctly from missing or unreported data. Accordingly, observed changes may reflect either: (1) true changes in service delivery or (2) incomplete, unreported or missing data. As the purpose of this analysis is to identify changes in actual service provision separate from missing data, we used PEPFAR's facility‐level data to categorize facilities according to their reporting consistency across eight quarters (2024 Q1−2025 Q4). The purpose of the facility categories is to establish a consistent longitudinal basis for comparison within each facility category to account for periods/quarters with gaps in reported data separate from changes in the number of services provided.

### 2.3 Facility Classification, Data Processing and Definitions

Facilities were categorized into seven groups based on reporting patterns:

- **Continuous facilities**: reported data in all eight quarters;
- **Intermittent facilities**: reported in both Q4 2024 and Q4 2025 but had gaps in other quarters;
- **Community services**: records without facility codes or identifiers (typically outreach programmes);
- **New in 2025**: began reporting during 2025 with no prior data in 2024;
- **Dropped in 2025**: facilities that reported data in Q4 2024, but did not report data in Q4 2025. This category distinguishes facilities that stopped reporting during 2025 from those who ceased reporting in 2024 or before the study period (“previously dropped”);
- **Previously dropped**: facilities that ceased reporting data sometime before Q4 2024;
- **Other**: facilities not fitting the above categories.

Classification was based on the presence or absence of at least one reported value for any indicator in a quarter, rather than indicator‐specific reporting. TX_NET_NEW (Quarterly increase or decrease in patients on treatment) was excluded, as it is a derived result rather than a directly reported measure.

Facility identifiers were constructed by combining sub‐national unit identifiers to ensure uniqueness. Community service sites were assigned unique identifiers at the lowest geographic level (typically a district).

### 2.4 Data Cleaning and Exclusions

Some facilities were identified as having changes in reported results suggestive of reporting errors, changes in reporting practices or methodological artefacts rather than true changes in service delivery and were excluded from the analysis. Details of the data cleaning procedures and their impact on the analysis are provided in the Supplementary Materials.

### 2.5 Outcome Measures

We assessed changes between 2024 and 2025 across key HIV service delivery indicators:

- Number of people receiving treatment, treatment initiations and return to treatment services;
- HIV testing and diagnoses;
- Prevention of mother‐to‐child transmission (PMTCT) programming; and
- PrEP initiations.

Analyses were conducted across all facility categories at the overall PEPFAR level and by country.

### 2.6 Human Resources for Health Analysis

We analysed PEPFAR Human Resources for Health (HRH) data to assess changes in workforce composition between 2024 and 2025. We excluded aggregate cadre‐level data from 2024 because these data were not directly comparable with 2025, and regional programme staff not assigned to specific countries. These exclusions represented only 5.32%of total staff in 2024 and 0.08% of staff across the 2 years, respectively.

### 2.7 Analysis

We calculated absolute and percentage changes between 2024 and 2025. Results are presented overall, by facility category and by country. For intermittent facilities, comparisons were based on Q4 2024 and Q4 2025 because complete annual data were unavailable.

### 2.8 Validity Analysis of 2025 Q1−Q3 Data and Facility Categorizations

To assess the reliability of the unofficial dataset and test the validity of the facility categorizations as a proxy for distinguishing true zero results from non‐reporting, we conducted two assessments.

Initially, we compared FY2025 Q4 results across both datasets at the facility level to determine the magnitude and frequency of differences between the two datasets.

Second, for each outcome indicator, we reported patterns across all eight quarters to identify changes in the number and proportion of facilities reporting null values. Among facilities with intermittent null values, we calculated each facility's average reported value during quarters in which positive values were recorded and summarized these distributions by quarter to assess whether the characteristics of facilities with null values differed between 2024 and 2025.

The full details and results for these tests are provided in the Supplementary Materials.

## 3 Results

### 3.1 Facility Reporting Patterns and Classification

**FIGURE 1:**
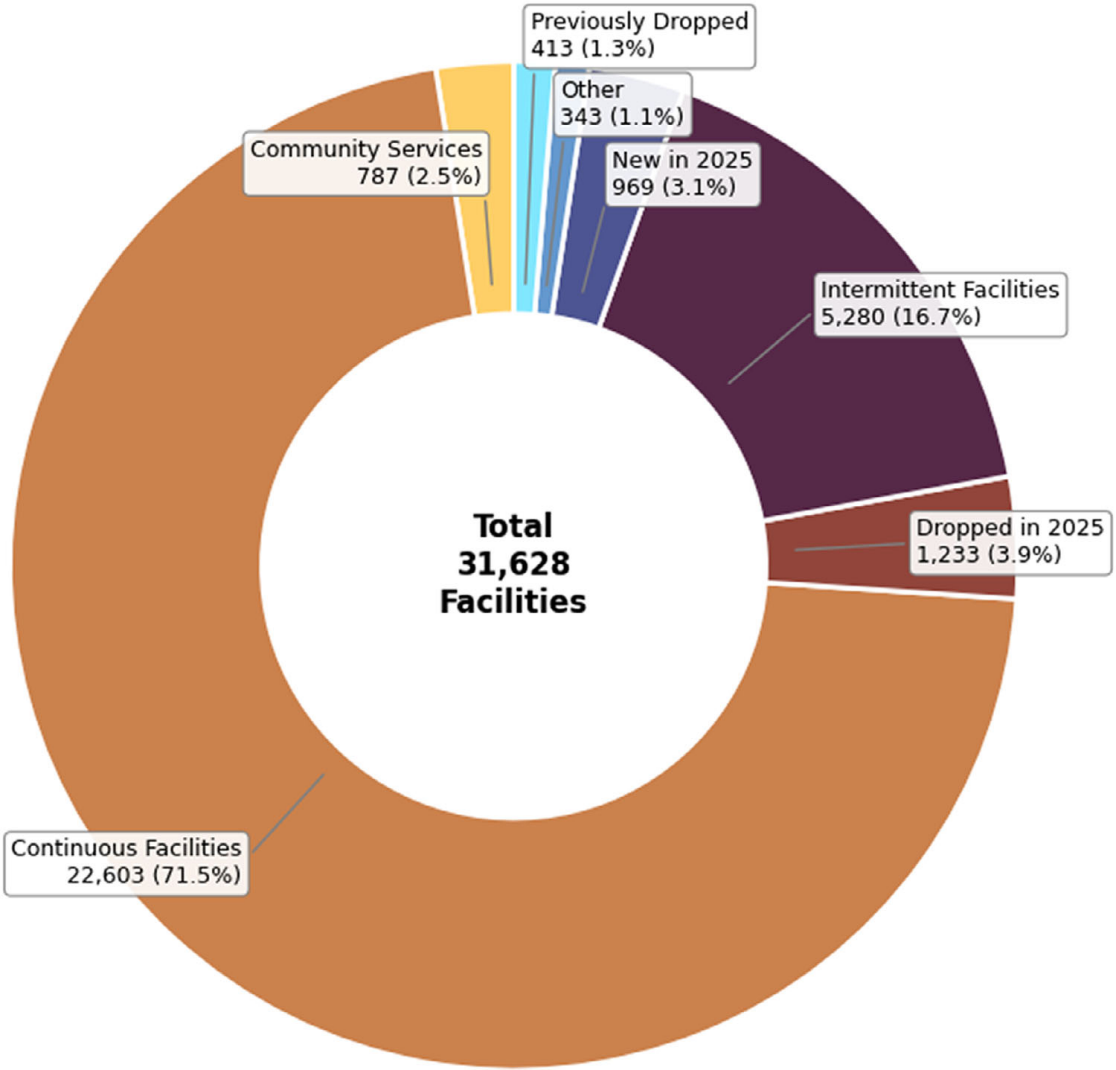
Continuous facilities: reported data every quarter from 2024Q1 to 2025Q4; Intermittent facilities: reported data in 2024Q4 and 2025Q4, but reported no data in one or more other quarters; Community services: sites in which the “Facility” code is blank; Dropped in 2025: reported data in 2024Q4, but reported no data as of 2025Q4; New in 2025: reported no data from 2024Q1 to 2024Q4, but began reporting sometime during 2025; Previously dropped: stopped reporting data sometime before 2024Q4; Other: facilities that did not fall into any of the above categories.

A total of 31,628 facilities and community service sites were included in the analysis (Figure 1). Most facilities (71.5%) reported continuously across all eight quarters from Q1 2024 to Q4 2025, while 16.7% were classified as intermittent reporters. Smaller proportions of facilities were classified as dropped in 2025 (3.9%) or newly reported in 2025 (3.1%), and community service sites accounted for 2.5% of all reporting units.

**FIGURE 2:**
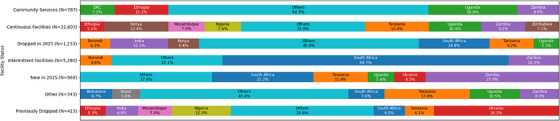
Facility categories by country facility count contribution.

Country‐level variation in reporting patterns is shown in Figure 2, with additional details provided in the Supplementary Materials.

South Africa was a noted outlier, accounting for 59.7% of all facilities classified as intermittent. This pattern is consistent with PEPFAR's “Central Support” reporting approach, whereby some results are reported annually rather than quarterly [22, 23]. The number of South African facilities reported through this mechanism increased from 1279 in Q4 2024 to 2278 in Q4 2025. Given the large contribution of South African facilities to the intermittent category, all analyses were conducted both including and excluding South Africa. Sensitivity analyses excluding South Africa are presented in the Supplementary Materials and available on GitHub [21].

### 3.2 Impact on the Number of People Accessing HIV Treatment

ART coverage remained relatively stable in continuous reporting facilities but declined among intermittent facilities and in areas where treatment sites were lost (Table 1).

**TABLE 1:** HIV treatment indicators (2024Q1−2024Q4).

HIV treatment initiations, current and returned patients by facility categorization (2024Q1−2025Q4)												
	2024					2025						
	Q1	Q2	Q3	Q4	Total	Q1	Q2	Q3	Q4	Total	Increase/decrease	Change (%)
**Current on treatment**												
Continuous facilities	15,505,668	15,452,717	15,479,644	15,634,073	15,634,073	15,625,336	15,272,109	15,430,064	15,678,650	15,678,650	44,577	0%
Dropped in 2025	355,159	363,280	366,059	441,822	441,822	96,771	34,195	12,593		0	−441,822	−100%
Intermittent facilities^a^	3,174,184	3,252,734	3,290,655	4,281,384	4,281,384	3,285,143	163,441	1,301,157	4,025,978	4,025,978	−255,406	−6%
New in 2025					0	32,451	38,470	51,290	370,846	370,846	370,846	inf%
Other	7635	2049	3424		0	7320	9489	7927	7988	7988	7988	inf%
Previously dropped	58,907	55,015	48,040		0					0	0	
All facilities	19,101,553	19,125,795	19,187,822	20,357,279	20,357,279	19,047,021	15,517,704	16,803,031	20,083,462	20,083,462	−273,817	−1%
**Newly initiated on treatment**												
Continuous facilities	318,260	333,932	310,760	307,876	1,270,828	283,345	247,564	264,940	271,012	1,066,861	−203,967	−16%
Dropped in 2025	10,032	9854	9714	16,656	46,256	3513	714	233		4460	−41,796	−90%
Intermittent facilities^a^	57,770	66,720	59,313	136,732	320,535	34,831	5709	21,012	85,075	146,627	−51,657	−38%
New in 2025					0	1647	1918	2446	15,743	21,754	21,754	inf%
Other	333	98	83		514	265	229	266	310	1070	556	108%
Previously dropped	1488	1302	699		3489					0	−3,489	−100%
All facilities	387,883	411,906	380,569	461,264	1,641,622	323,601	256,134	288,897	372,140	1,240,772	−400,850	−24%
**Patients returned to treatment**												
Continuous facilities	249,374	344,034	315,683	305,774	1,214,865	224,360	251,098	328,431	297,754	1,101,643	−113,222	−9%
Dropped in 2025	6481	7154	7390	7155	28,180	1221	319	591		2131	−26,049	−92%
Intermittent facilities^a^	91,440	115,823	107,872	105,038	420,173	47,033	3230	42,411	49,920	142,594	−55,118	−52%
New in 2025					0	840	1363	1521	2073	5797	5797	inf%
Other	272	34	110		416	167	266	382	917	1732	1316	316%
Previously dropped	1465	1694	1172		4331					0	−4331	−100%
All facilities	349,032	468,739	432,227	417,967	1,667,965	273,621	256,276	373,336	350,664	1,253,897	−414,068	−25%
**Viral load tests conducted**												
Continuous facilities	11,982,277	12,075,285	12,247,338	12,543,319	48,848,219	12,633,495	12,172,826	12,090,534	12,112,781	49,009,636	161,417	0%
Dropped in 2025	276,855	286,816	294,039	341,616	1,199,326	72,446	25,025	8057		105,528	−1,093,798	−91%
Intermittent facilities^a^	2,534,474	2,598,736	2,609,732	2,854,203	10,597,145	2,517,442	97,577	1,017,800	1,746,755	5,379,574	−1,107,448	−39%
New in 2025					0	19,961	24,134	32,717	302,304	379,116	379,116	inf%
Other	4168	1908	2723		8799	4478	6351	3,765	5419	20,013	11,214	127%
Previously dropped	45,640	43,927	40,683		130,250					0	−130,250	−100%
All facilities	14,843,414	15,006,672	15,194,515	15,739,138	60,783,739	15,247,822	12,325,913	13,152,873	14,167,259	54,893,867	−5,889,872	−10%

^a^ The increase/decrease and change (%) for intermittent facilities is calculated based only on 2024 Q4−2025 Q4 results. Increase/decrease and change (%) for all other categories of facilities are based on annual totals.

Across all facilities, the total number of people accessing HIV treatment declined by −273,817 (−1.3%) between Q4 2024 and Q4 2025. In continuous facilities, the number of people accessing treatment increased by 0.3% (44,577), although this represented 58.5% lower growth than observed in 2024 (Q1−Q4). In contrast, the number of people accessing treatment declined by −6.0% (*n* = −255,406) in intermittent facilities. Facilities classified as dropped supported 441,822 people in Q4 2024, and their treatment status in 2025 could not be determined. New facilities supported 370,846 people on treatment by Q4 2025.

Geographical analyses showed limited overlap between dropped and newly added facilities. Of 221 geographies in which treatment sites were lost during 2025, fewer than half (48%) also gained new facilities. Overall, the number of people accessing ART declined by −3% (*n* = −310,877) in geographies that lost treatment sites. Declines were greatest in areas where facilities were dropped without replacement (−7%, *n* = 116), whereas geographies with new facilities by no dropped sites experienced a small increase (−2%, *n* = −31,179).

### 3.3 Impact on the Number of People Tested, Diagnosed, Treatment Initiation and Retention Services

Substantial declines were observed across HIV testing, HIV diagnoses, treatment initiation and return‐to‐treatment (RTT) services (Table 2).

**TABLE 2:** HIV testing indicators (2024Q1−2024Q4).

HIV testing and diagnoses by facility categorization (2024Q1−2025Q4)												
	2024					2025					Increase/decrease	Change (%)
	Q1	Q2	Q3	Q4	Total	Q1	Q2	Q3	Q4	Total		
**HIV tests conducted**												
Community services	2,610,731	2,979,373	3,158,979	3,391,131	12,140,214	2,568,996	1,433,609	1,697,067	1,326,258	7,025,930	−5,114,284	−42%
Continuous facilities	13,288,871	14,581,178	14,709,657	15,478,190	58,057,896	14,191,119	12,408,470	13,810,087	14,164,158	54,573,834	−3,484,062	−6%
Dropped in 2025	169,372	188,137	187,241	317,256	862,006	107,647	24,836	10,410		142,893	−719,113	−83%
Intermittent facilities^a^	2,280,240	2,433,880	2,594,870	4,473,599	11,782,589	1,273,878	295,232	905,044	2,815,069	5,289,223	−1,658,530	−37%
New in 2025					0	97,476	109,542	145,035	929,070	1,281,123	1,281,123	inf%
Other	7539	4761	3629		15,929	9830	12,745	14,009	11,635	48,219	32,290	203%
Previously dropped	90,671	90,788	54,215		235,674					0	−235,674	−100%
All facilities	18,447,424	20,278,117	20,708,591	23,660,176	83,094,308	18,248,946	14,284,434	16,581,652	19,246,190	68,361,222	−14,733,086	−18%
**New HIV diagnoses**												
Community services	67,657	73,133	74,021	68,816	283,627	60,312	29,473	44,202	48,741	182,728	−100,899	−36%
Continuous facilities	264,551	275,923	250,091	250,997	1,041,562	232,495	217,016	226,768	228,585	904,864	−136,698	−13%
Dropped in 2025	6491	6357	5920	10,739	29,507	3194	814	296		4304	−25,203	−85%
Intermittent facilities^a^	56,262	62,714	55,970	115,797	290,743	28,659	5034	18,277	79,134	131,104	−36,663	−32%
New in 2025					0	1565	1751	2240	15,372	20,928	20,928	inf%
Other	261	88	101		450	235	189	275	241	940	490	109%
Previously dropped	1600	1373	675		3648					0	−3648	−100%
All facilities	396,822	419,588	386,778	446,349	1,649,537	326,460	254,277	292,058	372,073	1,244,868	−404,669	−25%

^a^ The increase/decrease and change (%) for intermittent facilities is calculated based only on 2024 Q4−2025 Q4 results. Increase/decrease and change (%) for all other categories of facilities are based on annual totals.

Across all facilities, PEPFAR‐supported HIV testing declined by −14.7 million tests between 2024 and 2025.

HIV diagnoses declined by −13% (*n* = −136,698 in continuous facilities, −36% (*n* = −100,899) in community service sites and −32% (*n* = −36,663) in intermittent facilities when comparing Q4 2024 with Q4 2025.

Treatment initiations also declined, decreasing by −16% (*n* = −203,967) in continuous facilities and −38% (*n* = −51,657) in intermittent facilities.

RTT services declined by −9% (*n* = −113,222) and −52% (*n* = −55,118), respectively. However, RTT services among intermittent facilities were highly variable by country, with 57% of intermittent facilities not reporting any RTT services in Q4 2025 compared to only 20% in Q4 2024.

Sensitivity analysis excluding South Africa showed only minor differences for continuous facilities, with HIV diagnoses declining by −12% and treatment initiations by −16%. Declines among intermittent facilities were attenuated (−30% for HIV diagnoses and −26% for treatment initiations) but remained substantially greater than those observed in continuous facilities.

### 3.4 Impact on PMTCT Service Delivery

Declines in PMTCT services were more pronounced among infants than among pregnant women (Table 3).

**TABLE 3:** PMTCT indicators (2024Q1−2024Q4).

PMTCT and early infant diagnoses and treatment by facility categorization (2024Q1−2025Q4)												
	2024					2025					Increase/decrease	Change (%)
	Q1	Q2	Q3	Q4	Total	Q1	Q2	Q3	Q4	Total		
**HIV tests in PMTCT programmes**												
Continuous facilities	3,072,481	3,377,786	3,205,331	3,261,208	12,916,806	3,178,630	3,086,078	3,263,709	3,329,278	12,857,695	−59,111	−0%
Dropped in 2025	18,772	23,462	23,330	24,893	90,457	12,090	1850	1299		15,239	−75,218	−83%
Intermittent facilities^a^	188,646	231,431	243,638	267,858	931,573	202,055	79,512	114,300	305,357	701,224	37,499	14%
New in 2025					0	26,361	32,181	34,187	46,032	138,761	138,761	inf%
Other	1178	1175	298		2651	2043	3,154	3521	3403	12,121	9470	357%
Previously dropped	29,655	30,391	25,015		85,061					0	−85,061	−100%
All facilities	3,310,732	3,664,245	3,497,612	3,553,959	14,026,548	3,421,179	3,202,775	3,417,016	3,684,070	13,725,040	−301,508	−2%
**New HIV diagnoses in PMTCT programmes**												
Continuous facilities	123,657	133,157	125,112	127,100	509,026	122,236	122,259	119,347	117,169	481,011	−28,015	−6%
Dropped in 2025	646	857	707	677	2887	340	67	77		484	−2403	−83%
Intermittent facilities^a^	22,142	25,544	22,990	23,919	94,595	11,477	1549	9067	49,607	71,700	25,688	107%
New in 2025					0	633	760	751	2556	4700	4700	inf%
Other	119	48	15		182	46	52	77	68	243	61	34%
Previously dropped	637	508	242		1387					0	−1387	−100%
All facilities	147,201	160,114	149,066	151,696	608,077	134,732	124,687	129,319	169,400	558,138	−49,939	−8%
PBFW on treatment												
Continuous facilities	123,135	131,975	125,012	131,333	511,455	123,273	121,451	120,170	117,956	482,850	−28,605	−6%
Dropped in 2025	632	847	657	5998	8134	288	67	76		431	−7703	−95%
Intermittent facilities^a^	22,113	25,496	22,871	33,017	103,497	17,331	1493	9039	20,297	48,160	−12,720	−39%
New in 2025					0	555	725	720	2518	4518	4518	inf%
Other	47	42	14		103	49	49	76	65	239	136	132%
Previously dropped	637	514	226		1377					0	−1377	−100%
All facilities	146,564	158,874	148,780	170,348	624,566	141,496	123,785	130,081	140,836	536,198	−88,368	−14%
**Early infant HIV diagnoses testing**												
Community services		2			2					0	−2	−100%
Continuous facilities	125,101	127,898	127,480	127,179	507,658	119,013	114,267	123,999	120,400	477,679	−29,979	−6%
Dropped in 2025	1388	1339	1309	1021	5057	302	50	60		412	−4645	−92%
Intermittent facilities^a^	37,390	37,435	37,192	33,274	145,291	22,392	1097	11,220	13,264	47,973	−20,010	−60%
New in 2025					0	304	398	421	476	1599	1599	inf%
Other	18	512	549		1079	542	524	553	580	2199	1120	104%
Previously dropped	324	339	165		828					0	−828	−100%
All facilities	164,221	167,525	166,695	161,474	659,915	142,553	116,336	136,253	134,720	529,862	−130,053	−20%
**Infants diagnosed HIV positive**												
Continuous facilities	1889	2199	2216	1997	8301	1763	1569	1900	2102	7334	−967	−12%
Dropped in 2025	14	16	16	11	57	9	2	2		13	−44	−77%
Intermittent facilities^a^	358	342	318	217	1235	299	17	115	149	580	−68	−31%
New in 2025					0	3	5	12	17	37	37	inf%
Other			2		2				1	1	−1	−50%
Previously dropped	1	1	3		5					0	−5	−100%
All facilities	2262	2558	2555	2225	9600	2074	1593	2029	2269	7965	−1635	−17%
**Infants diagnosed HIV positive within 2 months**												
Continuous facilities	853	997	1057	941	3848	779	650	756	949	3134	−714	−19%
Dropped in 2025	9	6	8	3	26	3		2		5	−21	−81%
Intermittent facilities^a^	180	181	171	104	636	128	8	55	73	264	−31	−30%
New in 2025					0	3	2	7	4	16	16	inf%
Other			2		2				1	1	−1	−50%
Previously dropped		1			1					0	−1	−100%
All facilities	1042	1185	1238	1048	4513	913	660	820	1027	3420	−1093	−24%
**Infants placed on HIV treatment**												
Continuous facilities	1662	1931	1887	1766	7246	1593	1311	1593	1871	6368	−878	−12%
Dropped in 2025	7	15	16	10	48	9	2	1		12	−36	−75%
Intermittent facilities^a^	293	287	247	181	1008	203	9	104	130	446	−51	−28%
New in 2025					0	2	5	9	14	30	30	inf%
Other					0				1	1	1	inf%
Previously dropped	1	4	1		6					0	−6	−100%
All facilities	1963	2237	2151	1957	8308	1807	1327	1707	2016	6857	−1451	−17%

^a^ The increase/decrease and change (%) for intermittent facilities is calculated based only on 2024 Q4−2025 Q4 results. Increase/decrease and change (%) for all other categories of facilities are based on annual totals. Abbreviations: PBFW, pregnant and breastfeeding women; PMTCT, prevention of mother‐to‐child transmission.

Testing and diagnoses among pregnant women remained relatively stable, declining by −2.1% and −8.2%, respectively.

In contrast, infant testing and diagnoses declined substantively. In continuous facilities, infant testing declined by −6% (*n* = −29,979) and infant HIV diagnoses by −12% (*n* = −967). Among intermittent facilities, comparing Q4 2024 to Q4 2025, infant testing declined by −60% (*n* = −20,010), infant HIV diagnoses by −31% (*n* = −68). Infant treatment initiations declined in line with infant diagnoses reductions.

Sensitivity analyses demonstrated that results from intermittent facilities were strongly influenced by South Africa, although the directionality and magnitude of this effect varied across PMTCT‐related indicators (Supplementary Materials).

### 3.5 Impact on PrEP

PrEP initiation declined substantially across all facility categories (Table 3).

Overall, PrEP initiations declined by −33%. In continuous facilities, PrEP initiations declined by −27% (*n* = −567,655), while Q4 2024 to Q4 2025 initiations in intermittent facilities declined by −63%.

### 3.6 Impact on Human Resources for Health

**FIGURE 3:**
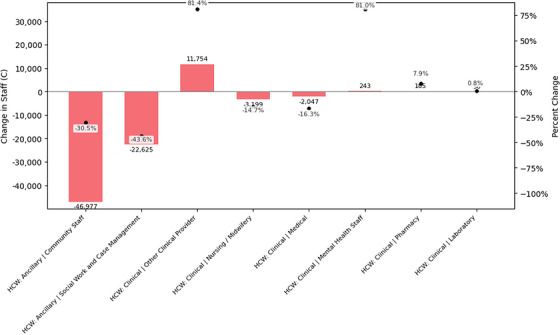
Change in employed staff by HCW category and cadre (2024−2025).

The PEPFAR‐supported workforce declined substantially between 2024 and 2025 (Figure 3).

Total staff numbers declined by −22% (*n* = −76,051) and −28% (*n* = −69,528) on a full‐time equivalent basis. By healthcare worker (HCW) category, ancillary HCWs (community staff, community‐based social workers and community case managers) declined by −34% (*n* = −69,602). Clinical HCWs (nurses, pharmacists, doctors/clinical officers, facility‐based social workers, lay counsellors and case managers) increased by 12% (*n* = 6992) [24].

Other technical, professional and data cadres decreased by −16% (*n* = −13,441). Overall, the combined number of ancillary and clinical HCWs declined by −24% (*n* = −62,541).

Country‐level patterns are presented in the Supplementary Materials.

### 3.7 Data Validity Analyses

Differences between the unofficial and official datasets were minimal. Changes to Q4 2025 data were confined to 132 South African facilities (<0.5% of all facilities reporting data in 2025 Q4).

Reporting patterns across indicators support the validity of facility classifications as a proxy for distinguishing between reductions in service delivery and non‐reporting.

Detailed methods and results of the validity analyses are provided in the Supplementary Materials.

## 4 Discussion

This is the first empirical analysis using programme data to evaluate changes in service delivery resulting from U.S. policy shifts. Declines were most substantial in HIV testing, HIV diagnoses, treatment initiation, prevention services and the PEPFAR‐supported health workforce, while the total number of people receiving ART remained relatively constant. Together, these findings suggest that HIV treatment programmes demonstrated short‐term resilience, whereas earlier stages of the care cascade were considerably more vulnerable to funding disruptions.

Interpretation of the relative stability of treatment coverage should be approached cautiously. The number of people receiving ART is likely a lagging indicator of programme performance. Governments responded to the foreign aid freeze to try to ensure dispensing of medicines was prioritized while other areas of the response by prioritizing continuity of treatment, while many programme activities were delayed, reduced or suspended. In addition, most people receiving ART were already established in care, and widespread implementation of multi‐month dispensing provided important protection against service interruptions. In Q1 2025, half (49.9%) of people receiving ART through PEPFAR were dispensed 6 months of medication at their most recent visit, while a further third (36%) received 3–5 months of medication. Consequently, many patients had sufficient medication to bridge the period of greatest programme disruption.

Despite this short‐term resilience, there are early indicators that treatment continuity may become increasingly difficult to sustain if disruptions persist. Growth in treatment coverage at continuously reporting facilities slowed by almost 60% compared with 2024, while treatment enrolment declined by 6% among intermittent facilities. Furthermore, the limited geographic overlap between dropped and newly reporting treatment sites and overall reductions in treatment enrolment in these geographies suggests that reductions in direct PEPFAR support were not systematically offset through replacement facilities or transitioned to other existing facilities. Instead, newly reporting sites appear to reflect changes in reporting or incorporation of existing facilities rather than expansion of treatment access.

The greatest vulnerability were observed in HIV testing, HIV diagnoses, treatment initiations, RTT services and prevention. Sustained reductions in HIV testing and case finding will inevitably reduce treatment initiation and ultimately affect the number of people receiving ART. Community service sites experienced particularly large declines in HIV diagnoses, highlighting the vulnerability of outreach services following reductions in donor support. If community‐based testing and outreach programmes, particularly those serving key populations, adolescents and other underserved groups, are not replaced through government financing or alternative donors, declines in case finding could lead to increased community viral load and HIV transmission, undermining progress towards the UNAIDS 95‐95‐95 targets and the targets established in the AFGHS MOUs with countries.

PMTCT findings require more cautious interpretation because several indicators were strongly influenced by reporting changes in South Africa. Nevertheless, reductions in early infant diagnosis were consistently observed among continuously reporting facilities and remained evident when South Africa was excluded from the analysis. Although broader epidemiological changes, including declining HIV prevalence among pregnant women and declining fertility, would be expected to reduce the number of HIV‐exposed infants requiring testing, reductions in infant testing should broadly parallel changes in the number of women living with HIV enrolled in care. The observed declines, therefore, warrant continued monitoring.

Prevention services also appear to have been disproportionately affected by the policy changes. The limited waiver in February 2025 excluded many prevention activities, including voluntary medical male circumcision (VMMC), gender‐based violence programmes, and key and priority population programming, and most PrEP services [25]. Facility‐level prevention indicators within MER are limited; however, the available data indicate substantial declines in PrEP initiation, while PEPFAR expenditure data show a 93% reduction in condom and lubricant programming [20]. Although interruptions to interventions such as VMMC may not immediately affect HIV incidence, they represent an important long‐term threat to epidemic control if not replaced through domestic financing or other partners.

These findings are broadly consistent with earlier empirical studies demonstrating widespread disruptions following the foreign aid freeze and with modelling studies projecting substantial increases in HIV morbidity and mortality if reductions in PEPFAR support were sustained [13, 14, 15, 16, 17, 18]. However, the observed programme data also highlight the adaptive responses of governments, health facilities, HCWs and patients that were not fully captured in early modelling exercises. These adaptations—including prioritization of treatment services—combined with pre‐existing practices like multi‐month dispensing of treatment likely explain why declines in ART coverage were smaller than many models projected. They also mirror the resilience in HIV treatment programmes during COVID‐19. Nevertheless, the substantial reductions observed in HIV testing, treatment initiation, prevention services and the health workforce are consistent with modelling predictions that prolonged disruption will ultimately result in increased HIV transmission, morbidity and mortality if these services are not restored.

The PEPFAR‐supported workforce also contracted substantially during 2025. Although some clinical cadres increased, these changes appear largely attributable to reclassification of staff rather than expansion of the workforce. Overall, the number of direct service delivery HCWs declined by 24%, including a 34% reduction in ancillary HCWs. These findings contrast with the AFGHS commitment to maintain all frontline health worker positions currently supported by PEPFAR [26]. These losses are also somewhat understated, as nearly 20,000 employed staff in the 2024 HRH data were excluded from the analysis as they lacked specific cadre data for comparison. Sustaining the HIV response will require continued investment not only in medicines, but also in the workforce required to deliver HIV prevention, testing, treatment and community services.

Several limitations should be considered when interpreting these findings. First, this analysis relied on unofficial Q1−Q3 2025 MER data, as only Q4 2025 data were included in the official public release. PEPFAR states the reason for withholding Q1−Q3 data as: “The U.S. government has greater confidence in fourth quarter data completeness following the transition of global health programs to the Department of State; earlier quarters experienced reporting and implementation challenges which limit data interpretability” [20]. We sought to minimize potential bias by restricting longitudinal analyses to continuously reporting facilities and community service sites, assessing intermittent facilities using Q4‐to‐Q4 comparisons only, and validating facility classifications using reporting patterns across indicators. Differences between the unofficial and official datasets for Q4 2025 data were minimal and entirely confined to a small minority of facilities in South Africa. This suggests that data cleaning processes for all other countries had already been completed by the time of the unofficial release and had only minor effects on the South Africa data, supporting the robustness of the analyses. Nevertheless, changes in reporting practices, data cleaning or de‐duplication may not have been fully captured and could have influenced some findings.

Second, MER data represent facilities receiving PEPFAR support and should not be interpreted as nationally representative measures of HIV service delivery. Although these facilities comprise a substantial proportion of HIV service delivery in many countries, assessing broader national impacts will require linkage with routine national health information systems and data from non‐PEPFAR‐supported facilities.

Overall, these findings suggest that the immediate impact of the 2025 disruptions was not uniform across HIV services. HIV treatment demonstrated considerable short‐term resilience, reflecting both government efforts and programme adaptations. However, substantial declines in HIV testing, treatment initiation, prevention services and the health workforce represent early warning signals that could undermine epidemic control if sustained. As countries assume greater responsibility for financing and delivering HIV services, careful monitoring of the entire HIV care cascade, not treatment coverage alone, will be essential to identify emerging gaps, prioritize investments and sustain progress towards ending the HIV epidemic.

## 5 Conclusions

This analysis provides the first comprehensive facility‐level assessment of PEPFAR programme performance following the 2025 U.S. foreign aid freeze. Although the number of people receiving ART remained relatively stable in continuously reporting facilities, this apparent resilience masked substantial declines across other parts of the HIV care cascade. HIV testing, HIV diagnoses, treatment initiation, prevention services and the PEPFAR‐supported workforce all declined, raising concerns about the sustainability of recent progress towards epidemic control if these disruptions persist.

These findings suggest that treatment coverage alone is an insufficient indicator of programme resilience. Monitoring the full HIV care cascade—including testing, prevention, treatment initiation, retention and the health workforce—will be essential as countries assume greater responsibility for HIV programmes. Sustained investment, transparent reporting and robust monitoring systems capable of tracking the full prevention, testing and treatment cascades will be critical to identify emerging service gaps, maintain accountability and prevent reversal of the gains achieved over the past two decades.

## Author Contributions

**Anna Grimsrud**: methodology, writing – review and editing. **Greg Millett**: writing – review and editing. **Jennifer Sherwood**: methodology, writing – review and editing. **Brian Honermann**: conceptualization, writing – original draft, methodology, validation, visualization, writing – review and editing, software, data curation, formal analysis, supervision. **Elise Lankiewicz**: methodology, writing – review and editing, visualization.

## Conflicts of Interest

The authors declare no conflicts of interest.

## Supporting information




**Supporting File**: jia270182‐sup‐0001‐SuppMat.docx

## Data Availability

The data that support the findings of this study are available on GitHub at https://github.com/amfAR‐PPO/PEPFAR_Impact_2025. These data were derived from the following resources available in the public domain: PEPFAR Panorama, https://data.pepfar.gov.
