# Supplementary material for: The Impact of the United States Foreign Aid Freeze on HIV Service Delivery in PEPFAR‐Supported Countries: A Facility‐Level Analysis of 2024–2025 Programme Data
Source: J Int AIDS Soc. 2026 Jul 27;29(8):e70182. doi: 10.1002/jia2.70182 (PMC13405359; doi:10.1002/jia2.70182)
Supplement: Supplementary file 1 — Supporting File: jia270182‐sup‐0001‐SuppMat.docx [file JIA2-29-e70182-s001.docx]

The impact of the United States foreign aid freeze on HIV service delivery in PEPFAR-supported countries: a facility-level analysis of 2024–2025 programme data

Supplemental Materials

Brian Honermann^1,✉^, Anna Grimsrud^2^, Elise Lankiewicz^1^, Jennifer Sherwood^1^, and Greg Millett^1^

^1^ amfAR, Andelson Office of Public Policy
^2^ International AIDS Society

^✉^ Correspondence: [Brian Honermann <brian.honermann@amfar.org>](mailto:brian.honermann@amfar.org)

# Overview

This document contains additional detailed analyses of PEPFAR’s data that are outside the scope of the main paper. All data for the paper and all tables, charts, and other materials are available in the project Github account.

For immediate ease of access, this document incorporates many of those tables directly, but more user friendly versions of these tables are obtainable in the Github repository.

# Changes from 2025 Q1-Q3 data set to final Q4 only data set

Comparisons between the 2025 Q4 data from the full year January release on the Panorama development dashboard and the final April Q4 only data release were performed to determine the reliability of the full-year dataset relative to the final dataset. The full results are in the associated excel file: “2025 Quarter 4 Data Changes.xlsx”.

In short, all changes between the January release and the April release were in South Africa. Changes were limited to 2.8% (132) of the 4,753 facilities in South Africa and 0.4% of the 31,746 facilities globally. Of the 132 facilities:

- 81% (107) were classified as intermittent facilities - for which the Q1-Q3 data play no part in our analysis;
- 11% (14) were classified as new facilities - for which the Q1-Q3 data are minimally involved in our analysis; and
- 8% (11) were classified as continuous sites - for which the Q1-Q3 data are involved in our analysis.

Of the 11 continuous sites, all had reporting changes for TX_CURR (Number of People on ART), but for which Q1-Q3 data play no role in our analysis. TX_CURR is a cumulative indicator rather than reflective of single quarter results and thus we only compare Q4 2024 to Q4 2025 data. Other indicators were more limited:

- HTS_TST (Total HIV tests): 2 continuous facilities with changes / total change +1,918 (0.003% of HTS_TST in 2025 in continuous facilities);
- HTS_TST_POS (Total HIV diagnoses): 1 continuous facility / +379 diagnoses (0.04% of HTS_TST_POS in 2025 in continuous facilities);
- PMTCT_ART (PBFW on Treatment): 2 continuous facilities / +335 (0.07% of PMTCT_ART in continuous facilities);
- PMTCT_STAT (HIV tests in PMTCT programs): 1 continuous facility / -3 (-0.00002% of PMTCT_STAT);
- PMTCT_STAT_POS (HIV diagnoses in PMTCT programs): 1 facility / -1 (-0.00002% of PMCTC_STAT_POS);
- TX_NEW (new on treatment): 2 facilities / +542 (0.05% of TX_NEW in continuous facilities);
- TX_PVLS: 2 facilities / +5,194 (0.01% of TX_PVLS in continuous facilities);

# Data Cleaning

Facility level PEPFAR data may have inconsistencies in reporting trends over time and needed to be excluded to ensure a proper apples-to-apples comparison of service delivery over the two-year time-period of the study. Primarily, these inconsistencies fall within a few buckets:

- **Unexplained Facility-Level Scale Changes**: Some facilities report Q4 2025 results for core indicators - like HIV tests conducted - that are an order of magnitude higher than their 2024 quarterly average.
- **Newly reporting previously unreported indicators**: Some facilities began reporting indicators for the first time only in Q3 or Q4 of 2025, despite a long history of reporting data into PEPFAR. While these could signal newly established services, for some indicators, it’s more likely that this reflects a change in reporting behavior rather than establishment of new services.
- **Individual Facility Indicator Scale Changes**: For some facilities, individual indicators show large expansions of services discordant from prior quarterly results that could reflect methodology changes or other inconsistencies in data reporting over the time period.

In some cases, changes in reporting patterns may be the result of changes in who is reporting. According to PEPFAR standards, implementing partners are only meant to report results for a facility if that facility receives some level of support from PEPFAR specifically for that service. So, while a facility may provide PMTCT services and have PEPFAR support for the treatment or testing programs, if the partner isn’t providing specific support for the PMTCT services, they would only report treatment and testing results for that facility while not reporting PMTCT related indicators. This seems likely to be the case in South Africa - the country with the greatest number of exclusions - where the National Department of Health took over reporting results for a large number of facilities that were previously reported only by implementing partners that were terminated in 2025.

In other cases such as TX_PVLS (Viral load testing) volume changes for facilities seem in many cases to likely be tied to facilities correcting reporting to be adherent to the actual indicator definition. The indicator requires reporting of the number of patients on treatment who have had a viral load test documented in the past 12 months. Many facilities appear to report quarterly test numbers in 2024 rather than 12 month coverage. While fidelity to the indicator definition may still vary from facility to facility and implementing partner to implementing partner, the purpose of this exclusion is to detect where a systematic change in reporting has taken place and exclude those data for the purposes of consistent comparison between the two time-periods.

We’ve implemented a three stage data cleaning effort to identify and exclude these abnormal results.

Stage 1: We identify facilities where any indicator experienced more than 1,000% growth between the 2024 quarterly average for that facility and the Q4 2025 result. For these facilities, all data for all indicators excluded.

Stage 2: For a subset of indicators: HTS_TST (HIV tests), PMTCT_EID (Infant HIV tests), PMTCT_STAT (HIV tests in PMTCT programs), TX_CURR (Number of people on treatment), TX_NEW (New initiations on treatment), and TX_PVLS (Viral load tests) we identify facilities that never reported a single result for these indicators in 2024, but reported a minimum of 10 in Q4 of 2025, despite their reporting of data for other indicators in other quarters.

Stage 3: For the same subset of indicators as stage 2, we identify facility data where facilities reported an average of 10 or more results per quarter in 2024, but reported a result at least 300% above that average in Q4 2025.

For facilities identified in Stage 2 or 3, we then exclude data for individual indicators as well as downstream indicators. We used the indicators referenced above because they are typically higher volume indicators and we have greater confidence using them as the basis for exclusion over lower volume indicators with a greater likelihood of true zero reporting. Downstream indicators are excluded as follows:

- HTS_TST_POS (HIV diagnoses) data are excluded if HTS_TST data are excluded;
- PMTCT_STAT_POS (HIV diagnoses in ANC) and PMTCT_ART (PBFW on treatment) are excluded if PMTCT_STAT data are excluded;
- PMTCT_HEI_POS (HIV diagnoses among infants), PMTCT_HEI_POS_2MO (HIV diagnoses among infants less than 2 months), and PMTCT_HEI_ART (Infants on treatment) are all excluded if PMTCT_EID data are excluded.

All data for excluded facilities are in the supplemental excel sheet “Facility Exclusions.xlsx”.

For stage 1 exclusions:

- 78 facilities were excluded, representing 0.25% of all facilities.
- By indicator:
  - **HTS_TST**: Facilities Excluded: 56; Mean 2024 Quarterly Result: 66; Mean 2025 Q4 Result: 2,052; Change Percentage: 2994%
  - **PMTCT_STAT**: Facilities Excluded: 7; Mean 2024 Quarterly Result: 45; Mean 2025 Q4 Result: 576; Change Percentage: 1241%
  - **TX_NEW**: Facilities Excluded: 3; Mean 2024 Quarterly Result: 42; Mean 2025 Q4 Result: 612; Change Percentage: 1456%
  - **TX_CURR**: Facilities Excluded: 5; Mean 2024 Quarterly Result: 72; Mean 2025 Q4 Result: 1,255; Change Percentage: 1588%
  - **TX_PVLS**: Facilities Excluded: 14; Mean 2024 Quarterly Result: 76; Mean 2025 Q4 Result: 1,344; Change Percentage: 1729%

For stage 2 exclusions:

- 1521 facilities had any data excluded. By indicator, exclusions were:
  - **HTS_TST**: Facilities Excluded: 149 with a total result of 54,696 (0.28% of total 2025Q4 results);
  - **HTS_TST_POS**: Facilities Excluded: 96 with a total result of 1,044 (0.28% of total 2025Q4 results);
  - **PMTCT_STAT**: Facilities Excluded: 1,133 with a total result of 138,185 (3.52% of total 2025Q4 results);
  - **PMTCT_STAT_POS**: Facilities Excluded: 1,040 with a total result of 35,305 (16.58% of total 2025Q4 results);
  - **PMTCT_ART**: Facilities Excluded: 1,079 with a total result of 46,302 (24.68% of total 2025Q4 results);
  - **TX_CURR**: Facilities Excluded: 253 with a total result of 158,175 (0.78% of total 2025Q4 results);
  - **TX_NEW**: Facilities Excluded: 124 with a total result of 9,638 (2.51% of total 2025Q4 results);
  - **TX_PVLS**: Facilities Excluded: 572 with a total result of 348,717 (2.40% of total 2025Q4 results);

For stage 3 exclusions:

- 687 facilities had any data excluded. By indicator, exclusions were:
  - **HTS_TST**: Facilities Excluded: 265 with a total result of 222,694 (1.14% of total 2025Q4 results);
  - **HTS_TST_POS**: Facilities Excluded: 253 with a total result of 4,634 (1.23% of total 2025Q4 results);
  - **PMTCT_STAT**: Facilities Excluded: 243 with a total result of 100,615 (2.56% of total 2025Q4 results);
  - **PMTCT_STAT_POS**: Facilities Excluded: 231 with a total result of 8,201 (3.85% of total 2025Q4 results);
  - **PMTCT_ART**: Facilities Excluded: 229 with a total result of 476 (0.25% of total 2025Q4 results);
  - **TX_CURR**: Facilities Excluded: 61 with a total result of 19,498 (0.10% of total 2025Q4 results);
  - **TX_NEW**: Facilities Excluded: 21 with a total result of 2,263 (0.59% of total 2025Q4 results);
  - **TX_PVLS**: Facilities Excluded: 129 with a total result of 38,180 (0.26% of total 2025Q4 results);

# Facility Categorizations and Null Results

The following table provides an analysis of the facility categorization’s suitability as a proxy for distinguishing between genuine drops in service delivery and the appearance of drops resultant only from non-reporting of data. Because PEPFAR’s data do not include true zero results, a null result for a facility in a given quarter may be reflective of a true zero or due to missing data.

For each indicator, facility categorization, and quarter across the time-period, we identify the total number of facilities that ever report a positive result for that indicator. For each quarter, we then identify the total number and percentage of facilities that have a null result (null-result facilities) to detect whether there are changes in the number and proportion of null-result facilities from 2024 to 2025.

Secondly, for each quarter for null-result facilities we additionally calculate the mean results each facility reports in quarters with positive results and then identify the median facility’s average quarterly results. This helps identify whether null-result facilities are generally small facilities for which true zero results are more plausible and whether there are changes in the size of null-result facilities over the time period.

If facility categorization is working well, we would expect to see the following for each facility categorization and indicator:

- **Continuous facilities**: The number and proportion of null-result facilities should remain relatively consistent across the 8 quarters, with a possible slight increase in 2025 resulting from genuine reductions in service delivery;
- **Community Services**: Increases in the number, proportion, and size of non-result community “sites” that is consistent with a reduction of services delivered through community modalities previously funded by PEPFAR;
- **Intermittent facilities**: Relatively high variability in the numbers, proportions, and median facility results for non-result facilities in Q1 to Q3. Q4 results should largely be in-line with those of continuous sites.
- **Dropped in 2025**: Variable levels on non-report facilities in 2024 across quarters, but increasing percentages of non-report facilities in 2025 reaching 100% by 2025 Q4;
- **New in 2025**: 100% non-report facilities in all of 2024 with reductions in non-report facilities across 2025 and Q4 non-result facility percentages similar to continuous sites;
- **Previously dropped**: Will reach 100% non-report facilities by Q4 2024 and remain there for all of 2025;
- **Other**: Variable rates across quarters with unclear pattern or trend.

Intermittent facilities are inclusive of facilities that only began reporting data sometime during 2024 resulting in disproportionately high non-result facility proportions in Q1 and Q2 of 2024. To account for this, we used PEPFAR 2023 facility level data to identify intermittent facilities that only began reporting data in 2024 and created a sub-category of “Intermittent excluding new” to account for these facilities. The table below includes the full “Intermittent facilities” results as well as the sub-category results. This does not affect any of the other facility categorizations.

Finally, on an indicator by indicator basis, we would expect to see some directional changes from 2024 to 2025 for continuous facilities, intermittent facilities, and community services if there have been genuine reductions in specific services. For instance, for HIV self-test kit distribution (HTS_SELF), we would expect to see increases in the number, proportion, and size of null-report facilities in 2025 relative to 2024 consistent with self-test kit distribution activities being de-prioriritized in 2025. We should also see alignment in the directionality of change for inter-related indicators. For instance, if continuous facilities have stable HIV testing results, we would anticipate that new diagnoses and new initiations on treatment would be follow that pattern. Likewise, movement in the number and proportion of non-report facilities for intermittent sites across those three indicators should be directionally correlated.

The table below shows the results which largely align with expectations, with some exceptions. Patients returned to treatment (RTT) and Viral load tests conducted both show increased proportions of null-result facilities specifically for intermittent facilities. For RTT, this pattern holds true whether South Africa data are included or excluded, though viral load tests numbers are more in-line with 2024 results when excluding South Africa. There’s insufficient information to make conclusions on whether this suggests that RTT services were actually discontinued in these sites or just went unrecorded. We don’t discuss the viral load testing results in the main paper, but the that the null-report facilities are primarily in South Africa suggests that those differences are likely non-reporting of data rather than true halting of viral load services. South Africa largely funds all its own viral load testing in accordance with their natioanl guidelines.

| Number and percent of facilities reporting no data by indicator and facility categorization | | | | | | | | | | | | | | | | | | | | | | | | | |
| --- | --- | --- | --- | --- | --- | --- | --- | --- | --- | --- | --- | --- | --- | --- | --- | --- | --- | --- | --- | --- | --- | --- | --- | --- | --- |
|  | Total Facilities | 2024 | | | | | | | | 2025 | | | | | | | | Median facility's mean quarterly non-null results for null facilities (2024) | | | | Median facility's mean quarterly non-null results for null facilities (2025) | | | |
|  |  | Q1 | (%) | Q2 | (%) | Q3 | (%) | Q4 | (%) | Q1 | (%) | Q2 | (%) | Q3 | (%) | Q4 | (%) | Q1 | Q2 | Q3 | Q4 | Q1 | Q2 | Q3 | Q4 |
| HIV Self Test Kit Distribution | | | | | | | | | | | | | | | | | | | | | | | | | |
| Community Services | 585 | 97 | (17%) | 90 | (15%) | 72 | (12%) | 80 | (14%) | 112 | (19%) | 193 | (33%) | 293 | (50%) | 301 | (51%) | 75 | 62 | 70 | 70 | 151 | 204 | 319 | 304 |
| Continuous Facilities | 14,626 | 5,416 | (37%) | 4,818 | (33%) | 4,921 | (34%) | 5,250 | (36%) | 6,944 | (47%) | 8,002 | (55%) | 7,127 | (49%) | 6,880 | (47%) | 30 | 30 | 25 | 26 | 29 | 31 | 30 | 31 |
| Dropped in 2025 | 210 | 100 | (48%) | 78 | (37%) | 92 | (44%) | 71 | (34%) | 120 | (57%) | 181 | (86%) | 185 | (88%) | 210 | (100%) | 29 | 24 | 20 | 24 | 31 | 50 | 46 | 50 |
| Intermittent Excluding New | 1,550 | 556 | (36%) | 485 | (31%) | 484 | (31%) | 493 | (32%) | 696 | (45%) | 1,523 | (98%) | 1,300 | (84%) | 927 | (60%) | 33 | 32 | 28 | 32 | 35 | 58 | 51 | 51 |
| Intermittent Facilities | 1,993 | 999 | (50%) | 903 | (45%) | 781 | (39%) | 708 | (36%) | 899 | (45%) | 1,822 | (91%) | 1,510 | (76%) | 1,137 | (57%) | 28 | 28 | 25 | 25 | 30 | 48 | 43 | 40 |
| New in 2025 | 134 | 134 | (100%) | 134 | (100%) | 134 | (100%) | 134 | (100%) | 96 | (72%) | 93 | (69%) | 67 | (50%) | 44 | (33%) | 21 | 21 | 21 | 21 | 22 | 18 | 15 | 9 |
| Other | 30 | 28 | (93%) | 23 | (77%) | 26 | (87%) | 30 | (100%) | 17 | (57%) | 22 | (73%) | 27 | (90%) | 25 | (83%) | 22 | 24 | 24 | 23 | 21 | 18 | 21 | 22 |
| Previously Dropped | 79 | 14 | (18%) | 20 | (25%) | 53 | (67%) | 79 | (100%) | 79 | (100%) | 79 | (100%) | 79 | (100%) | 79 | (100%) | 33 | 18 | 38 | 46 | 46 | 46 | 46 | 46 |
| HIV Tests Conducted | | | | | | | | | | | | | | | | | | | | | | | | | |
| Community Services | 669 | 26 | (4%) | 20 | (3%) | 24 | (4%) | 24 | (4%) | 64 | (10%) | 109 | (16%) | 135 | (20%) | 131 | (20%) | 71 | 71 | 110 | 134 | 213 | 416 | 409 | 376 |
| Continuous Facilities | 21,640 | 207 | (1%) | 209 | (1%) | 209 | (1%) | 170 | (1%) | 192 | (1%) | 346 | (2%) | 306 | (1%) | 282 | (1%) | 33 | 54 | 69 | 74 | 42 | 88 | 93 | 79 |
| Dropped in 2025 | 997 | 257 | (26%) | 213 | (21%) | 207 | (21%) | 49 | (5%) | 556 | (56%) | 852 | (85%) | 890 | (89%) | 997 | (100%) | 45 | 48 | 43 | 27 | 70 | 86 | 94 | 91 |
| Intermittent Excluding New | 3,934 | 1,277 | (32%) | 1,236 | (31%) | 1,252 | (32%) | 36 | (1%) | 1,501 | (38%) | 3,657 | (93%) | 3,001 | (76%) | 224 | (6%) | 759 | 816 | 792 | 8 | 681 | 540 | 525 | 220 |
| Intermittent Facilities | 4,899 | 2,241 | (46%) | 1,829 | (37%) | 1,467 | (30%) | 47 | (1%) | 1,536 | (31%) | 3,703 | (76%) | 3,047 | (62%) | 241 | (5%) | 300 | 406 | 616 | 16 | 666 | 532 | 520 | 200 |
| New in 2025 | 866 | 866 | (100%) | 866 | (100%) | 866 | (100%) | 866 | (100%) | 364 | (42%) | 314 | (36%) | 267 | (31%) | 5 | (1%) | 108 | 108 | 108 | 108 | 69 | 48 | 36 | 27 |
| Other | 154 | 102 | (66%) | 104 | (68%) | 116 | (75%) | 154 | (100%) | 69 | (45%) | 80 | (52%) | 69 | (45%) | 101 | (66%) | 66 | 68 | 70 | 70 | 56 | 44 | 36 | 47 |
| Previously Dropped | 277 | 58 | (21%) | 69 | (25%) | 162 | (58%) | 277 | (100%) | 277 | (100%) | 277 | (100%) | 277 | (100%) | 277 | (100%) | 22 | 17 | 58 | 72 | 72 | 72 | 72 | 72 |
| New HIV Diagnoses | | | | | | | | | | | | | | | | | | | | | | | | | |
| Community Services | 660 | 38 | (6%) | 25 | (4%) | 31 | (5%) | 41 | (6%) | 74 | (11%) | 137 | (21%) | 157 | (24%) | 153 | (23%) | 4 | 4 | 4 | 4 | 17 | 17 | 22 | 18 |
| Continuous Facilities | 21,334 | 2,399 | (11%) | 2,204 | (10%) | 2,379 | (11%) | 2,482 | (12%) | 2,729 | (13%) | 3,046 | (14%) | 2,886 | (14%) | 2,911 | (14%) | 2 | 2 | 2 | 2 | 2 | 2 | 2 | 2 |
| Dropped in 2025 | 814 | 264 | (32%) | 257 | (32%) | 260 | (32%) | 163 | (20%) | 502 | (62%) | 713 | (88%) | 742 | (91%) | 814 | (100%) | 2 | 2 | 2 | 2 | 3 | 4 | 4 | 4 |
| Intermittent Excluding New | 3,837 | 1,478 | (39%) | 1,423 | (37%) | 1,430 | (37%) | 280 | (7%) | 2,144 | (56%) | 3,644 | (95%) | 3,029 | (79%) | 692 | (18%) | 17 | 18 | 18 | 2 | 16 | 13 | 14 | 3 |
| Intermittent Facilities | 4,743 | 2,384 | (50%) | 2,129 | (45%) | 1,850 | (39%) | 508 | (11%) | 2,368 | (50%) | 3,905 | (82%) | 3,288 | (69%) | 960 | (20%) | 6 | 7 | 10 | 2 | 13 | 12 | 12 | 2 |
| New in 2025 | 680 | 680 | (100%) | 680 | (100%) | 680 | (100%) | 680 | (100%) | 379 | (56%) | 314 | (46%) | 284 | (42%) | 148 | (22%) | 3 | 3 | 3 | 3 | 3 | 2 | 2 | 2 |
| Other | 97 | 73 | (75%) | 81 | (84%) | 85 | (88%) | 97 | (100%) | 48 | (49%) | 62 | (64%) | 45 | (46%) | 69 | (71%) | 2 | 3 | 2 | 3 | 3 | 2 | 2 | 2 |
| Previously Dropped | 177 | 30 | (17%) | 41 | (23%) | 109 | (62%) | 177 | (100%) | 177 | (100%) | 177 | (100%) | 177 | (100%) | 177 | (100%) | 3 | 2 | 3 | 4 | 4 | 4 | 4 | 4 |
| PBFW on Treatment | | | | | | | | | | | | | | | | | | | | | | | | | |
| Continuous Facilities | 19,040 | 3,979 | (21%) | 3,817 | (20%) | 3,904 | (21%) | 3,871 | (20%) | 4,005 | (21%) | 4,367 | (23%) | 4,031 | (21%) | 4,270 | (22%) | 2 | 2 | 2 | 2 | 2 | 2 | 2 | 2 |
| Dropped in 2025 | 252 | 104 | (41%) | 104 | (41%) | 120 | (48%) | 72 | (29%) | 166 | (66%) | 235 | (93%) | 235 | (93%) | 252 | (100%) | 2 | 2 | 2 | 1 | 2 | 2 | 2 | 2 |
| Intermittent Excluding New | 2,302 | 731 | (32%) | 679 | (29%) | 690 | (30%) | 362 | (16%) | 1,128 | (49%) | 2,246 | (98%) | 1,813 | (79%) | 1,261 | (55%) | 2 | 2 | 2 | 2 | 4 | 5 | 4 | 4 |
| Intermittent Facilities | 2,996 | 1,425 | (48%) | 1,275 | (43%) | 1,128 | (38%) | 666 | (22%) | 1,415 | (47%) | 2,543 | (85%) | 2,092 | (70%) | 1,551 | (52%) | 2 | 2 | 2 | 2 | 2 | 4 | 3 | 3 |
| New in 2025 | 433 | 433 | (100%) | 433 | (100%) | 433 | (100%) | 433 | (100%) | 264 | (61%) | 216 | (50%) | 206 | (48%) | 117 | (27%) | 2 | 2 | 2 | 2 | 2 | 2 | 2 | 1 |
| Other | 43 | 34 | (79%) | 33 | (77%) | 36 | (84%) | 43 | (100%) | 28 | (65%) | 22 | (51%) | 22 | (51%) | 26 | (60%) | 2 | 2 | 2 | 2 | 2 | 4 | 1 | 1 |
| Previously Dropped | 62 | 14 | (23%) | 15 | (24%) | 31 | (50%) | 62 | (100%) | 62 | (100%) | 62 | (100%) | 62 | (100%) | 62 | (100%) | 2 | 3 | 2 | 3 | 3 | 3 | 3 | 3 |
| Early Infant HIV Diagnoses Testing | | | | | | | | | | | | | | | | | | | | | | | | | |
| Community Services | 1 | 1 | (100%) | 0 | (0%) | 1 | (100%) | 1 | (100%) | 1 | (100%) | 1 | (100%) | 1 | (100%) | 1 | (100%) | 2 |  | 2 | 2 | 2 | 2 | 2 | 2 |
| Continuous Facilities | 17,439 | 4,712 | (27%) | 4,334 | (25%) | 4,200 | (24%) | 4,290 | (25%) | 4,435 | (25%) | 5,282 | (30%) | 4,746 | (27%) | 4,606 | (26%) | 2 | 2 | 2 | 2 | 2 | 2 | 2 | 2 |
| Dropped in 2025 | 186 | 57 | (31%) | 68 | (37%) | 70 | (38%) | 63 | (34%) | 116 | (62%) | 174 | (94%) | 175 | (94%) | 186 | (100%) | 2 | 2 | 2 | 2 | 2 | 2 | 2 | 3 |
| Intermittent Excluding New | 2,087 | 574 | (28%) | 480 | (23%) | 527 | (25%) | 644 | (31%) | 1,084 | (52%) | 2,053 | (98%) | 1,837 | (88%) | 1,509 | (72%) | 2 | 2 | 2 | 2 | 4 | 5 | 5 | 5 |
| Intermittent Facilities | 2,548 | 1,035 | (41%) | 917 | (36%) | 826 | (32%) | 897 | (35%) | 1,321 | (52%) | 2,374 | (93%) | 2,100 | (82%) | 1,776 | (70%) | 2 | 2 | 2 | 2 | 3 | 4 | 4 | 4 |
| New in 2025 | 200 | 200 | (100%) | 200 | (100%) | 200 | (100%) | 200 | (100%) | 122 | (61%) | 105 | (52%) | 90 | (45%) | 79 | (40%) | 2 | 2 | 2 | 2 | 1 | 1 | 1 | 1 |
| Other | 21 | 17 | (81%) | 17 | (81%) | 16 | (76%) | 21 | (100%) | 12 | (57%) | 10 | (48%) | 12 | (57%) | 12 | (57%) | 1 | 1 | 1 | 2 | 1 | 1 | 1 | 2 |
| Previously Dropped | 54 | 10 | (19%) | 16 | (30%) | 20 | (37%) | 54 | (100%) | 54 | (100%) | 54 | (100%) | 54 | (100%) | 54 | (100%) | 3 | 2 | 4 | 4 | 4 | 4 | 4 | 4 |
| Infants Diagnosed HIV Positive | | | | | | | | | | | | | | | | | | | | | | | | | |
| Continuous Facilities | 5,906 | 4,594 | (78%) | 4,383 | (74%) | 4,402 | (75%) | 4,530 | (77%) | 4,619 | (78%) | 4,825 | (82%) | 4,571 | (77%) | 4,468 | (76%) | 1 | 1 | 1 | 1 | 1 | 1 | 1 | 1 |
| Dropped in 2025 | 37 | 27 | (73%) | 27 | (73%) | 26 | (70%) | 26 | (70%) | 30 | (81%) | 35 | (95%) | 35 | (95%) | 37 | (100%) | 1 | 1 | 1 | 1 | 1 | 1 | 1 | 1 |
| Intermittent Excluding New | 690 | 443 | (64%) | 449 | (65%) | 469 | (68%) | 540 | (78%) | 542 | (79%) | 689 | (100%) | 623 | (90%) | 597 | (87%) | 1 | 1 | 1 | 1 | 1 | 1 | 1 | 1 |
| Intermittent Facilities | 756 | 509 | (67%) | 513 | (68%) | 521 | (69%) | 592 | (78%) | 589 | (78%) | 742 | (98%) | 676 | (89%) | 651 | (86%) | 1 | 1 | 1 | 1 | 1 | 1 | 1 | 1 |
| New in 2025 | 21 | 21 | (100%) | 21 | (100%) | 21 | (100%) | 21 | (100%) | 18 | (86%) | 18 | (86%) | 12 | (57%) | 7 | (33%) | 1 | 1 | 1 | 1 | 1 | 1 | 1 | 1 |
| Other | 2 | 2 | (100%) | 2 | (100%) | 1 | (50%) | 2 | (100%) | 2 | (100%) | 2 | (100%) | 2 | (100%) | 1 | (50%) | 2 | 2 | 1 | 2 | 2 | 2 | 2 | 2 |
| Previously Dropped | 4 | 3 | (75%) | 3 | (75%) | 1 | (25%) | 4 | (100%) | 4 | (100%) | 4 | (100%) | 4 | (100%) | 4 | (100%) | 1 | 1 | 1 | 1 | 1 | 1 | 1 | 1 |
| HIV Tests in PMTCT Programs | | | | | | | | | | | | | | | | | | | | | | | | | |
| Continuous Facilities | 19,757 | 507 | (3%) | 345 | (2%) | 368 | (2%) | 317 | (2%) | 273 | (1%) | 466 | (2%) | 416 | (2%) | 515 | (3%) | 54 | 14 | 23 | 5 | 5 | 15 | 18 | 54 |
| Dropped in 2025 | 310 | 71 | (23%) | 68 | (22%) | 54 | (17%) | 46 | (15%) | 127 | (41%) | 280 | (90%) | 289 | (93%) | 310 | (100%) | 6 | 4 | 4 | 4 | 53 | 45 | 46 | 45 |
| Intermittent Excluding New | 2,329 | 284 | (12%) | 279 | (12%) | 240 | (10%) | 240 | (10%) | 1,092 | (47%) | 2,185 | (94%) | 1,677 | (72%) | 239 | (10%) | 5 | 4 | 4 | 4 | 42 | 50 | 59 | 10 |
| Intermittent Facilities | 3,139 | 1,094 | (35%) | 774 | (25%) | 400 | (13%) | 259 | (8%) | 1,118 | (36%) | 2,226 | (71%) | 1,707 | (54%) | 261 | (8%) | 29 | 21 | 7 | 4 | 40 | 49 | 58 | 11 |
| New in 2025 | 680 | 680 | (100%) | 680 | (100%) | 680 | (100%) | 680 | (100%) | 262 | (39%) | 232 | (34%) | 205 | (30%) | 9 | (1%) | 33 | 33 | 33 | 33 | 21 | 20 | 20 | 8 |
| Other | 63 | 45 | (71%) | 44 | (70%) | 50 | (79%) | 63 | (100%) | 31 | (49%) | 25 | (40%) | 28 | (44%) | 33 | (52%) | 33 | 33 | 37 | 33 | 24 | 25 | 7 | 20 |
| Previously Dropped | 88 | 15 | (17%) | 16 | (18%) | 39 | (44%) | 88 | (100%) | 88 | (100%) | 88 | (100%) | 88 | (100%) | 88 | (100%) | 26 | 32 | 48 | 118 | 118 | 118 | 118 | 118 |
| New HIV Diagnoses in PMTCT Programs | | | | | | | | | | | | | | | | | | | | | | | | | |
| Continuous Facilities | 19,055 | 4,033 | (21%) | 3,754 | (20%) | 3,898 | (20%) | 3,885 | (20%) | 4,005 | (21%) | 4,325 | (23%) | 4,050 | (21%) | 4,219 | (22%) | 2 | 2 | 2 | 2 | 2 | 2 | 2 | 2 |
| Dropped in 2025 | 236 | 82 | (35%) | 83 | (35%) | 96 | (41%) | 85 | (36%) | 136 | (58%) | 218 | (92%) | 219 | (93%) | 236 | (100%) | 1 | 1 | 1 | 1 | 2 | 2 | 2 | 2 |
| Intermittent Excluding New | 2,150 | 568 | (26%) | 526 | (24%) | 500 | (23%) | 449 | (21%) | 1,323 | (62%) | 2,093 | (97%) | 1,665 | (77%) | 596 | (28%) | 2 | 2 | 2 | 2 | 6 | 6 | 4 | 2 |
| Intermittent Facilities | 2,850 | 1,268 | (44%) | 1,120 | (39%) | 896 | (31%) | 748 | (26%) | 1,606 | (56%) | 2,378 | (83%) | 1,936 | (68%) | 866 | (30%) | 2 | 2 | 2 | 2 | 4 | 4 | 3 | 2 |
| New in 2025 | 478 | 478 | (100%) | 478 | (100%) | 478 | (100%) | 478 | (100%) | 286 | (60%) | 257 | (54%) | 245 | (51%) | 126 | (26%) | 2 | 2 | 2 | 2 | 2 | 2 | 2 | 1 |
| Other | 40 | 30 | (75%) | 29 | (72%) | 34 | (85%) | 40 | (100%) | 25 | (62%) | 20 | (50%) | 17 | (42%) | 24 | (60%) | 2 | 2 | 2 | 2 | 2 | 4 | 2 | 2 |
| Previously Dropped | 60 | 13 | (22%) | 15 | (25%) | 28 | (47%) | 60 | (100%) | 60 | (100%) | 60 | (100%) | 60 | (100%) | 60 | (100%) | 2 | 3 | 3 | 4 | 4 | 4 | 4 | 4 |
| PrEP Initiations | | | | | | | | | | | | | | | | | | | | | | | | | |
| Continuous Facilities | 14,557 | 4,064 | (28%) | 3,348 | (23%) | 3,158 | (22%) | 3,008 | (21%) | 2,942 | (20%) | 4,793 | (33%) | 4,827 | (33%) | 4,447 | (31%) | 4 | 4 | 3 | 3 | 3 | 5 | 5 | 5 |
| Dropped in 2025 | 374 | 101 | (27%) | 82 | (22%) | 89 | (24%) | 74 | (20%) | 167 | (45%) | 302 | (81%) | 367 | (98%) | 374 | (100%) | 6 | 6 | 3 | 3 | 9 | 15 | 21 | 20 |
| Intermittent Excluding New | 2,229 | 497 | (22%) | 433 | (19%) | 407 | (18%) | 399 | (18%) | 444 | (20%) | 2,165 | (97%) | 1,593 | (71%) | 1,405 | (63%) | 4 | 3 | 4 | 3 | 3 | 12 | 11 | 11 |
| Intermittent Facilities | 2,723 | 991 | (36%) | 869 | (32%) | 708 | (26%) | 573 | (21%) | 596 | (22%) | 2,504 | (92%) | 1,917 | (70%) | 1,725 | (63%) | 4 | 4 | 3 | 3 | 3 | 10 | 9 | 8 |
| New in 2025 | 182 | 182 | (100%) | 182 | (100%) | 182 | (100%) | 182 | (100%) | 126 | (69%) | 108 | (59%) | 77 | (42%) | 40 | (22%) | 5 | 5 | 5 | 5 | 4 | 3 | 2 | 2 |
| Other | 55 | 49 | (89%) | 50 | (91%) | 50 | (91%) | 55 | (100%) | 22 | (40%) | 44 | (80%) | 42 | (76%) | 44 | (80%) | 30 | 28 | 21 | 26 | 9 | 30 | 38 | 38 |
| Previously Dropped | 88 | 24 | (27%) | 19 | (22%) | 42 | (48%) | 88 | (100%) | 88 | (100%) | 88 | (100%) | 88 | (100%) | 88 | (100%) | 5 | 7 | 12 | 14 | 14 | 14 | 14 | 14 |
| Current on Treatment | | | | | | | | | | | | | | | | | | | | | | | | | |
| Continuous Facilities | 21,677 | 151 | (1%) | 103 | (0%) | 57 | (0%) | 54 | (0%) | 54 | (0%) | 236 | (1%) | 84 | (0%) | 80 | (0%) | 32 | 32 | 9 | 23 | 66 | 325 | 200 | 113 |
| Dropped in 2025 | 701 | 124 | (18%) | 111 | (16%) | 109 | (16%) | 30 | (4%) | 315 | (45%) | 607 | (87%) | 658 | (94%) | 701 | (100%) | 32 | 31 | 31 | 29 | 159 | 93 | 98 | 101 |
| Intermittent Excluding New | 3,880 | 1,273 | (33%) | 1,256 | (32%) | 1,237 | (32%) | 28 | (1%) | 1,320 | (34%) | 3,703 | (95%) | 2,958 | (76%) | 63 | (2%) | 436 | 444 | 454 | 4 | 426 | 537 | 530 | 67 |
| Intermittent Facilities | 4,760 | 2,153 | (45%) | 1,839 | (39%) | 1,482 | (31%) | 69 | (1%) | 1,375 | (29%) | 3,751 | (79%) | 2,994 | (63%) | 73 | (2%) | 164 | 228 | 330 | 3 | 394 | 529 | 522 | 58 |
| New in 2025 | 661 | 661 | (100%) | 661 | (100%) | 661 | (100%) | 661 | (100%) | 290 | (44%) | 221 | (33%) | 166 | (25%) | 5 | (1%) | 36 | 36 | 36 | 36 | 50 | 54 | 58 | 1 |
| Other | 68 | 42 | (62%) | 47 | (69%) | 49 | (72%) | 68 | (100%) | 37 | (54%) | 28 | (41%) | 24 | (35%) | 33 | (49%) | 42 | 65 | 63 | 56 | 68 | 38 | 30 | 48 |
| Previously Dropped | 130 | 11 | (8%) | 31 | (24%) | 55 | (42%) | 130 | (100%) | 130 | (100%) | 130 | (100%) | 130 | (100%) | 130 | (100%) | 90 | 76 | 76 | 146 | 146 | 146 | 146 | 146 |
| Newly Initiated on Treatment | | | | | | | | | | | | | | | | | | | | | | | | | |
| Continuous Facilities | 21,174 | 1,964 | (9%) | 1,722 | (8%) | 1,815 | (9%) | 1,952 | (9%) | 2,030 | (10%) | 2,449 | (12%) | 2,231 | (11%) | 2,299 | (11%) | 2 | 2 | 2 | 2 | 2 | 2 | 2 | 2 |
| Dropped in 2025 | 639 | 176 | (28%) | 164 | (26%) | 172 | (27%) | 118 | (18%) | 358 | (56%) | 575 | (90%) | 611 | (96%) | 639 | (100%) | 3 | 2 | 2 | 2 | 5 | 5 | 6 | 6 |
| Intermittent Excluding New | 3,772 | 1,446 | (38%) | 1,411 | (37%) | 1,398 | (37%) | 219 | (6%) | 1,683 | (45%) | 3,625 | (96%) | 2,966 | (79%) | 1,267 | (34%) | 24 | 26 | 26 | 2 | 23 | 16 | 16 | 11 |
| Intermittent Facilities | 4,649 | 2,323 | (50%) | 2,110 | (45%) | 1,835 | (39%) | 429 | (9%) | 1,883 | (41%) | 3,877 | (83%) | 3,188 | (69%) | 1,495 | (32%) | 9 | 10 | 15 | 2 | 18 | 14 | 15 | 8 |
| New in 2025 | 597 | 597 | (100%) | 597 | (100%) | 597 | (100%) | 597 | (100%) | 349 | (58%) | 253 | (42%) | 235 | (39%) | 105 | (18%) | 3 | 3 | 3 | 3 | 3 | 3 | 3 | 2 |
| Other | 56 | 41 | (73%) | 38 | (68%) | 46 | (82%) | 56 | (100%) | 35 | (62%) | 27 | (48%) | 23 | (41%) | 34 | (61%) | 3 | 4 | 4 | 4 | 5 | 3 | 3 | 2 |
| Previously Dropped | 94 | 11 | (12%) | 18 | (19%) | 36 | (38%) | 94 | (100%) | 94 | (100%) | 94 | (100%) | 94 | (100%) | 94 | (100%) | 6 | 4 | 5 | 8 | 8 | 8 | 8 | 8 |
| Viral Load Tests Conducted | | | | | | | | | | | | | | | | | | | | | | | | | |
| Continuous Facilities | 20,413 | 364 | (2%) | 250 | (1%) | 162 | (1%) | 159 | (1%) | 152 | (1%) | 216 | (1%) | 161 | (1%) | 148 | (1%) | 15 | 9 | 7 | 7 | 9 | 25 | 16 | 21 |
| Dropped in 2025 | 640 | 96 | (15%) | 83 | (13%) | 81 | (13%) | 33 | (5%) | 270 | (42%) | 546 | (85%) | 607 | (95%) | 640 | (100%) | 14 | 16 | 21 | 13 | 176 | 67 | 68 | 77 |
| Intermittent Excluding New | 3,578 | 1,035 | (29%) | 1,001 | (28%) | 982 | (27%) | 54 | (2%) | 1,070 | (30%) | 3,418 | (96%) | 2,685 | (75%) | 1,024 | (29%) | 218 | 230 | 232 | 4 | 216 | 377 | 368 | 814 |
| Intermittent Facilities | 4,296 | 1,753 | (41%) | 1,558 | (36%) | 1,299 | (30%) | 162 | (4%) | 1,164 | (27%) | 3,516 | (82%) | 2,756 | (64%) | 1,060 | (25%) | 86 | 98 | 132 | 3 | 182 | 360 | 344 | 781 |
| New in 2025 | 549 | 549 | (100%) | 549 | (100%) | 549 | (100%) | 549 | (100%) | 271 | (49%) | 230 | (42%) | 166 | (30%) | 20 | (4%) | 22 | 22 | 22 | 22 | 21 | 18 | 18 | 2 |
| Other | 52 | 35 | (67%) | 37 | (71%) | 38 | (73%) | 52 | (100%) | 28 | (54%) | 20 | (38%) | 23 | (44%) | 25 | (48%) | 71 | 60 | 45 | 50 | 72 | 56 | 148 | 72 |
| Previously Dropped | 119 | 15 | (13%) | 31 | (26%) | 47 | (39%) | 119 | (100%) | 119 | (100%) | 119 | (100%) | 119 | (100%) | 119 | (100%) | 59 | 5 | 10 | 102 | 102 | 102 | 102 | 102 |
| Patients Returned to Treatment | | | | | | | | | | | | | | | | | | | | | | | | | |
| Continuous Facilities | 18,551 | 5,892 | (32%) | 4,792 | (26%) | 4,694 | (25%) | 4,940 | (27%) | 5,675 | (31%) | 5,831 | (31%) | 6,525 | (35%) | 6,902 | (37%) | 2 | 2 | 2 | 2 | 2 | 2 | 2 | 2 |
| Dropped in 2025 | 425 | 147 | (35%) | 154 | (36%) | 134 | (32%) | 135 | (32%) | 301 | (71%) | 403 | (95%) | 403 | (95%) | 425 | (100%) | 2 | 2 | 2 | 2 | 4 | 4 | 4 | 4 |
| Intermittent Excluding New | 2,441 | 356 | (15%) | 334 | (14%) | 330 | (14%) | 311 | (13%) | 1,270 | (52%) | 2,384 | (98%) | 1,687 | (69%) | 1,452 | (59%) | 2 | 2 | 2 | 2 | 21 | 20 | 18 | 21 |
| Intermittent Facilities | 3,043 | 958 | (31%) | 880 | (29%) | 726 | (24%) | 617 | (20%) | 1,587 | (52%) | 2,743 | (90%) | 1,942 | (64%) | 1,732 | (57%) | 3 | 3 | 2 | 2 | 13 | 15 | 12 | 14 |
| New in 2025 | 331 | 331 | (100%) | 331 | (100%) | 331 | (100%) | 331 | (100%) | 231 | (70%) | 153 | (46%) | 124 | (37%) | 104 | (31%) | 3 | 3 | 3 | 3 | 3 | 2 | 3 | 2 |
| Other | 32 | 24 | (75%) | 26 | (81%) | 25 | (78%) | 32 | (100%) | 22 | (69%) | 19 | (59%) | 15 | (47%) | 21 | (66%) | 4 | 4 | 4 | 4 | 4 | 4 | 4 | 4 |
| Previously Dropped | 83 | 20 | (24%) | 25 | (30%) | 32 | (39%) | 83 | (100%) | 83 | (100%) | 83 | (100%) | 83 | (100%) | 83 | (100%) | 2 | 3 | 3 | 8 | 8 | 8 | 8 | 8 |

# Facility categorization by country

This table provides the full breakdown by country of how each facility was categorized.

| Facility Categorization by Country | | | | | | | | |
| --- | --- | --- | --- | --- | --- | --- | --- | --- |
|  | Community Services | Continuous Facilities | Dropped in 2025 | Intermittent Facilities | New in 2025 | Other | Previously Dropped | Total |
| Angola | 4 (15%) | 19 (70%) | 1 (4%) | 1 (4%) | 1 (4%) | 1 (4%) | 0 (0%) | 27 |
| Benin | 5 (23%) | 17 (77%) | 0 (0%) | 0 (0%) | 0 (0%) | 0 (0%) | 0 (0%) | 22 |
| Botswana | 21 (12%) | 111 (61%) | 21 (12%) | 1 (1%) | 1 (1%) | 23 (13%) | 4 (2%) | 182 |
| Brazil | 0 (0%) | 17 (59%) | 2 (7%) | 3 (10%) | 7 (24%) | 0 (0%) | 0 (0%) | 29 |
| Burkina Faso | 5 (13%) | 31 (79%) | 1 (3%) | 0 (0%) | 0 (0%) | 2 (5%) | 0 (0%) | 39 |
| Burundi | 14 (3%) | 0 (0%) | 78 (17%) | 349 (76%) | 0 (0%) | 13 (3%) | 8 (2%) | 462 |
| Cameroon | 10 (3%) | 347 (96%) | 4 (1%) | 0 (0%) | 0 (0%) | 0 (0%) | 1 (0%) | 362 |
| Colombia | 1 (33%) | 1 (33%) | 1 (33%) | 0 (0%) | 0 (0%) | 0 (0%) | 0 (0%) | 3 |
| Côte d'Ivoire | 28 (5%) | 514 (93%) | 4 (1%) | 2 (0%) | 0 (0%) | 2 (0%) | 3 (1%) | 553 |
| Democratic Republic of the Congo | 57 (9%) | 485 (73%) | 4 (1%) | 112 (17%) | 0 (0%) | 2 (0%) | 4 (1%) | 664 |
| Dominican Republic | 11 (20%) | 23 (42%) | 9 (16%) | 1 (2%) | 9 (16%) | 2 (4%) | 0 (0%) | 55 |
| El Salvador | 1 (2%) | 33 (53%) | 4 (6%) | 15 (24%) | 8 (13%) | 1 (2%) | 0 (0%) | 62 |
| Eswatini | 4 (1%) | 165 (62%) | 7 (3%) | 78 (29%) | 8 (3%) | 4 (1%) | 2 (1%) | 268 |
| Ethiopia | 88 (6%) | 1,147 (79%) | 54 (4%) | 108 (7%) | 24 (2%) | 11 (1%) | 22 (2%) | 1,454 |
| Ghana | 3 (2%) | 119 (97%) | 0 (0%) | 1 (1%) | 0 (0%) | 0 (0%) | 0 (0%) | 123 |
| Guatemala | 1 (2%) | 26 (50%) | 11 (21%) | 2 (4%) | 11 (21%) | 1 (2%) | 0 (0%) | 52 |
| Haiti | 10 (6%) | 80 (44%) | 52 (29%) | 30 (17%) | 3 (2%) | 3 (2%) | 3 (2%) | 181 |
| Honduras | 1 (2%) | 28 (61%) | 7 (15%) | 6 (13%) | 0 (0%) | 0 (0%) | 4 (9%) | 46 |
| India | 6 (2%) | 170 (43%) | 149 (38%) | 26 (7%) | 6 (2%) | 7 (2%) | 28 (7%) | 392 |
| Indonesia | 5 (3%) | 135 (88%) | 2 (1%) | 0 (0%) | 11 (7%) | 1 (1%) | 0 (0%) | 154 |
| Jamaica | 1 (4%) | 16 (67%) | 1 (4%) | 0 (0%) | 5 (21%) | 1 (4%) | 0 (0%) | 24 |
| Kazakhstan | 2 (29%) | 5 (71%) | 0 (0%) | 0 (0%) | 0 (0%) | 0 (0%) | 0 (0%) | 7 |
| Kenya | 29 (1%) | 3,024 (89%) | 79 (2%) | 209 (6%) | 38 (1%) | 16 (0%) | 12 (0%) | 3,407 |
| Kyrgyzstan | 4 (13%) | 22 (73%) | 0 (0%) | 0 (0%) | 0 (0%) | 0 (0%) | 4 (13%) | 30 |
| Lao People's Democratic Republic | 1 (4%) | 15 (65%) | 1 (4%) | 2 (9%) | 4 (17%) | 0 (0%) | 0 (0%) | 23 |
| Lesotho | 10 (4%) | 100 (43%) | 6 (3%) | 106 (46%) | 6 (3%) | 0 (0%) | 3 (1%) | 231 |
| Liberia | 1 (3%) | 32 (82%) | 6 (15%) | 0 (0%) | 0 (0%) | 0 (0%) | 0 (0%) | 39 |
| Malawi | 17 (2%) | 753 (90%) | 34 (4%) | 15 (2%) | 3 (0%) | 8 (1%) | 6 (1%) | 836 |
| Mali | 3 (12%) | 23 (88%) | 0 (0%) | 0 (0%) | 0 (0%) | 0 (0%) | 0 (0%) | 26 |
| Mozambique | 11 (1%) | 1,701 (91%) | 8 (0%) | 60 (3%) | 39 (2%) | 17 (1%) | 29 (2%) | 1,865 |
| Myanmar | 5 (10%) | 0 (0%) | 19 (38%) | 19 (38%) | 0 (0%) | 3 (6%) | 4 (8%) | 50 |
| Namibia | 14 (3%) | 377 (85%) | 15 (3%) | 9 (2%) | 7 (2%) | 2 (0%) | 20 (5%) | 444 |
| Nepal | 6 (6%) | 27 (25%) | 34 (32%) | 16 (15%) | 4 (4%) | 20 (19%) | 0 (0%) | 107 |
| Nicaragua | 1 (8%) | 0 (0%) | 10 (77%) | 0 (0%) | 1 (8%) | 0 (0%) | 1 (8%) | 13 |
| Nigeria | 35 (2%) | 1,721 (94%) | 2 (0%) | 11 (1%) | 17 (1%) | 0 (0%) | 51 (3%) | 1,837 |
| Panama | 1 (3%) | 17 (53%) | 7 (22%) | 5 (16%) | 1 (3%) | 1 (3%) | 0 (0%) | 32 |
| Papua New Guinea | 0 (0%) | 11 (100%) | 0 (0%) | 0 (0%) | 0 (0%) | 0 (0%) | 0 (0%) | 11 |
| Peru | 1 (50%) | 1 (50%) | 0 (0%) | 0 (0%) | 0 (0%) | 0 (0%) | 0 (0%) | 2 |
| Philippines | 1 (1%) | 70 (88%) | 3 (4%) | 0 (0%) | 3 (4%) | 2 (2%) | 1 (1%) | 80 |
| Rwanda | 5 (2%) | 223 (78%) | 6 (2%) | 29 (10%) | 12 (4%) | 10 (3%) | 2 (1%) | 287 |
| Senegal | 7 (23%) | 22 (71%) | 0 (0%) | 1 (3%) | 1 (3%) | 0 (0%) | 0 (0%) | 31 |
| Sierra Leone | 0 (0%) | 85 (87%) | 5 (5%) | 2 (2%) | 4 (4%) | 1 (1%) | 1 (1%) | 98 |
| South Africa | 31 (1%) | 1,089 (23%) | 182 (4%) | 3,153 (67%) | 205 (4%) | 26 (1%) | 27 (1%) | 4,713 |
| South Sudan | 27 (20%) | 80 (59%) | 3 (2%) | 21 (16%) | 1 (1%) | 2 (1%) | 1 (1%) | 135 |
| Tajikistan | 3 (4%) | 70 (93%) | 1 (1%) | 1 (1%) | 0 (0%) | 0 (0%) | 0 (0%) | 75 |
| Tanzania | 31 (1%) | 3,018 (88%) | 114 (3%) | 60 (2%) | 110 (3%) | 61 (2%) | 25 (1%) | 3,419 |
| Thailand | 1 (2%) | 11 (20%) | 26 (48%) | 0 (0%) | 14 (26%) | 2 (4%) | 0 (0%) | 54 |
| Togo | 4 (12%) | 29 (85%) | 1 (3%) | 0 (0%) | 0 (0%) | 0 (0%) | 0 (0%) | 34 |
| Trinidad and Tobago | 1 (2%) | 2 (5%) | 29 (66%) | 0 (0%) | 0 (0%) | 11 (25%) | 1 (2%) | 44 |
| Uganda | 146 (5%) | 2,456 (86%) | 65 (2%) | 84 (3%) | 54 (2%) | 36 (1%) | 1 (0%) | 2,842 |
| Ukraine | 24 (3%) | 354 (47%) | 53 (7%) | 143 (19%) | 63 (8%) | 14 (2%) | 108 (14%) | 759 |
| Viet Nam | 10 (6%) | 100 (60%) | 45 (27%) | 3 (2%) | 4 (2%) | 1 (1%) | 3 (2%) | 166 |
| Zambia | 69 (2%) | 2,083 (68%) | 38 (1%) | 556 (18%) | 270 (9%) | 28 (1%) | 17 (1%) | 3,061 |
| Zimbabwe | 10 (1%) | 1,598 (93%) | 29 (2%) | 40 (2%) | 14 (1%) | 8 (0%) | 17 (1%) | 1,716 |

# Sensitivity analysis of South Africa’s effect on outcomes and conclusions

Due to South African facilities making up ~60% of all intermittent facilities, we ran all analyses with and without South Africa included. Visual and table outputs excluding South Africa are in the paper’s project repository on Github.

Below is a table showing the impact of South Africa on the results by facility categorization and indicator. Intermittent facilities see the largest swings in terms of the Q4 2024 to Q4 2025 change in results, but the direction of change goes in both positive and negative directions.

Removing South Africa substantially shrinks the number and proportion of facilities classified as intermittent. With South Africa included, continuous facilities represent 71.5% of sites (22,603) and intermittent sites are 16.7% (5,280). Excluding South Africa, continuous facilities increase to 79.9% of sites (21,514) while intermittent sites drop to 7.9% (2,127).

For testing services, continuous facilities saw minimal change (-6.0% to -5.8% with and without South Africa, respectively) but intermittent facilities saw a +20.6% change (-37.1% to -16.5%), while still performing worse than continuous facilities. HIV diagnoses were similar, continuous sites performed marginally better (+0.8% from -13.1% to -12.3%) while intermittent facilities increased 9.7% (-31.7% to -22.0%).

Treatment numbers were less affected, with continuous facilities seeing no proportional change in the outcomes, while intermitten facilities were marginally better off (-6.0% to -4.2% with and without South Africa, respectively).

PMTCT programming varied the most for intermittent sites comparing results with and without South Africa. As was described above regarding Data Cleaning, South Africa’s PMTCT data were particularly erratic, likely due to change-overs in who was reporting data for some facilities, but not all such changes were likely caught by our data cleaning algorithms. For HIV testing in PMTCT programs (PMTCT_STAT), intermittent sites did 19.5% worse when South Africa was not included (+14.0% to -5.5%), while continuous facilities changed marginally (+0.1%). HIV diagnoses in PMTCT programs also were massively affected going from an increase of 107.4% in intermittent facilities to -7.5%, while continuous facilities improved marginally from -5.5% to -4.6%. Other PMTCT related programs for intermittent facilities saw significant changes as well: Early infant diagnoses: +45.1% (-60.1% to -15.0%); infants diagnosed positive: +31.3% (-31.3% to 0.0%);

PrEP initiations in intermittent sites performed somewhat worse when South Africa was excluded, dropping from -62.9% with South Africa to -76.4% in 2024Q4 to 2025Q4 results comparisons.

The overall significance of South Africa on overall results is primarily in intermittent facilities, with continuous facilities seeing only minor changes across all indicators. However, excluding South Africa from inclusion in some cases just leads to other individual countries being the driving force in outcomes for intermittent facilities for that indicator. For instance, for patients being returned to treatment (TX_RTT), with and without South Africa data being included, 2024Q4 to 2025Q4 results swing from a drop of -52.5% to an increase of 26.7% - a swing of +79.2 percentage points. However, the outcome with South Africa excluded is now based ultimately on a total numerical change of an increase in TX_RTT from 10,546 in 2024Q4 to 13,363 in 2025Q4 (+2,817). This is largely driven by Mozambique where for intermittent facilities, TX_RTT increased quarter over quarter by +3,248 (+332%) and Zambia (+1,543 / +231%). Without Mozambique, the overall quarter over quarter change would move from +26.7% to -3.9% - a swing of 30.6%. Without Mozabmique and Zambia, the ultimate change is -21.8%, or -48.5% from just excluding South Africa alone.

Ultimately, intermittent facilities have more stable overall results, while intermittent facilities are more variable in their outcomes and by country.

| Impact of South Africa on Change (%): Included vs. Excluded | | | |
| --- | --- | --- | --- |
|  | Change (%) incl. ZAF | Change (%) excl. ZAF | Difference |
| HIV Tests Conducted | | | |
| Community Services | −42.1% | −41.1% | 1.0% |
| Continuous Facilities | −6.0% | −5.8% | 0.2% |
| Dropped in 2025 | −83.4% | −78.7% | 4.7% |
| Intermittent Facilities* | −37.1% | −16.5% | 20.6% |
| New in 2025 | inf% | inf% |  |
| Other | 202.7% | 199.6% | −3.1% |
| Previously Dropped | −100.0% | −100.0% | 0.0% |
| New HIV Diagnoses | | | |
| Community Services | −35.6% | −33.8% | 1.8% |
| Continuous Facilities | −13.1% | −12.3% | 0.8% |
| Dropped in 2025 | −85.4% | −81.7% | 3.7% |
| Intermittent Facilities* | −31.7% | −22.0% | 9.7% |
| New in 2025 | inf% | inf% |  |
| Other | 108.9% | 110.6% | 1.7% |
| Previously Dropped | −100.0% | −100.0% | 0.0% |
| Current on Treatment | | | |
| Continuous Facilities | 0.3% | 0.3% | 0.0% |
| Dropped in 2025 | −100.0% | −100.0% | 0.0% |
| Intermittent Facilities* | −6.0% | −4.2% | 1.8% |
| New in 2025 | inf% | inf% |  |
| Other | inf% | inf% |  |
| Previously Dropped |  |  |  |
| Newly Initiated on Treatment | | | |
| Continuous Facilities | −16.0% | −15.5% | 0.5% |
| Dropped in 2025 | −90.4% | −88.1% | 2.3% |
| Intermittent Facilities* | −37.8% | −19.4% | 18.4% |
| New in 2025 | inf% | inf% |  |
| Other | 108.2% | 110.8% | 2.6% |
| Previously Dropped | −100.0% | −100.0% | 0.0% |
| Patients Returned to Treatment | | | |
| Continuous Facilities | −9.3% | −7.1% | 2.2% |
| Dropped in 2025 | −92.4% | −92.8% | −0.4% |
| Intermittent Facilities* | −52.5% | 26.7% | 79.2% |
| New in 2025 | inf% | inf% |  |
| Other | 316.3% | 325.0% | 8.7% |
| Previously Dropped | −100.0% | −100.0% | 0.0% |
| Viral Load Tests Conducted | | | |
| Continuous Facilities | 0.3% | 0.3% | −0.1% |
| Dropped in 2025 | −91.2% | −91.2% | 0.0% |
| Intermittent Facilities* | −38.8% | −22.1% | 16.7% |
| New in 2025 | inf% | inf% |  |
| Other | 127.4% | 129.0% | 1.6% |
| Previously Dropped | −100.0% | −100.0% | 0.0% |
| HIV Tests in PMTCT Programs | | | |
| Continuous Facilities | −0.5% | −0.3% | 0.1% |
| Dropped in 2025 | −83.2% | −82.8% | 0.4% |
| Intermittent Facilities* | 14.0% | −5.5% | −19.5% |
| New in 2025 | inf% | inf% |  |
| Other | 357.2% | 362.4% | 5.2% |
| Previously Dropped | −100.0% | −100.0% | 0.0% |
| New HIV Diagnoses in PMTCT Programs | | | |
| Continuous Facilities | −5.5% | −4.6% | 0.9% |
| Dropped in 2025 | −83.2% | −81.4% | 1.8% |
| Intermittent Facilities* | 107.4% | −7.5% | −114.9% |
| New in 2025 | inf% | inf% |  |
| Other | 33.5% | 35.8% | 2.2% |
| Previously Dropped | −100.0% | −100.0% | 0.0% |
| PBFW on Treatment | | | |
| Continuous Facilities | −5.6% | −3.9% | 1.7% |
| Dropped in 2025 | −94.7% | −83.6% | 11.1% |
| Intermittent Facilities* | −38.5% | −10.2% | 28.3% |
| New in 2025 | inf% | inf% |  |
| Other | 132.0% | 139.0% | 7.0% |
| Previously Dropped | −100.0% | −100.0% | 0.0% |
| Early Infant HIV Diagnoses Testing | | | |
| Community Services | −100.0% | −100.0% | 0.0% |
| Continuous Facilities | −5.9% | −5.2% | 0.7% |
| Dropped in 2025 | −91.9% | −78.3% | 13.6% |
| Intermittent Facilities* | −60.1% | −15.0% | 45.1% |
| New in 2025 | inf% | inf% |  |
| Other | 103.8% | 64.4% | −39.4% |
| Previously Dropped | −100.0% | −100.0% | 0.0% |
| Infants Diagnosed HIV Positive | | | |
| Continuous Facilities | −11.6% | −12.1% | −0.4% |
| Dropped in 2025 | −77.2% | −64.9% | 12.3% |
| Intermittent Facilities* | −31.3% | 0.0% | 31.3% |
| New in 2025 | inf% | inf% |  |
| Other | −50.0% | −50.0% | 0.0% |
| Previously Dropped | −100.0% | −100.0% | 0.0% |
| Infants Diagnosed HIV Positive within 2 Months | | | |
| Continuous Facilities | −18.6% | −18.5% | 0.0% |
| Dropped in 2025 | −80.8% | −66.7% | 14.1% |
| Intermittent Facilities* | −29.8% | −7.4% | 22.4% |
| New in 2025 | inf% | inf% |  |
| Other | −50.0% | −50.0% | 0.0% |
| Previously Dropped | −100.0% | −100.0% | 0.0% |
| Infants Placed on HIV Treatment | | | |
| Continuous Facilities | −12.1% | −12.3% | −0.2% |
| Dropped in 2025 | −75.0% | −63.6% | 11.4% |
| Intermittent Facilities* | −28.2% | 2.1% | 30.3% |
| New in 2025 | inf% | inf% |  |
| Other | inf% | inf% |  |
| Previously Dropped | −100.0% | −100.0% | 0.0% |
| PrEP Initiations | | | |
| Continuous Facilities | −27.3% | −28.4% | −1.1% |
| Dropped in 2025 | −76.3% | −75.1% | 1.2% |
| Intermittent Facilities* | −62.9% | −76.4% | −13.5% |
| New in 2025 | inf% | inf% |  |
| Other | 611.4% | 828.4% | 216.9% |
| Previously Dropped | −100.0% | −100.0% | 0.0% |
| Voluntary Medical Male Circumcisions | | | |
| Continuous Facilities | −100.0% | −100.0% | 0.0% |
| Dropped in 2025 | −100.0% | −100.0% | 0.0% |
| Intermittent Facilities* | −100.0% | −100.0% | 0.0% |
| Other | −100.0% |  |  |
| Previously Dropped | −100.0% | −100.0% | 0.0% |
| * Intermittent Facilities: change is based on 2024 Q4 to 2025 Q4 only. All other facility categories reflect full-year changes (2024 annual total vs. 2025 annual total). | | | |
